# A three boron doped B/O/N multi-resonant TADF emitter for improved reverse intersystem crossing rate and efficient pure blue organic light-emitting diodes†

**DOI:** 10.1039/d5sc03560k

**Published:** 2025-07-10

**Authors:** Sen Wu, Dongyang Chen, Mathilde Seinfeld, Aidan P. McKay, David B. Cordes, Xiaohong Zhang, Eli Zysman-Colman

**Affiliations:** a Organic Semiconductor Centre, EaStCHEM School of Chemistry, University of St Andrews St Andrews, Fife KY16 9ST UK eli.zysman-colman@st-andrews.ac.uk +44-1334 463808 +44-1334 463826; b Institute of Functional Nano & Soft Materials (FUNSOM), Joint International Research Laboratory of Carbon-Based Functional Materials and Devices, Soochow University Suzhou Jiangsu 215123 P. R. China xiaohong_zhang@suda.edu.cn; c Jiangsu Key Laboratory of Advanced Negative Carbon Technologies, Soochow University Suzhou 215123 Jiangsu P. R. China

## Abstract

Multiresonant thermally activated delayed fluorescence (MR-TADF) compounds exhibit significant potential as emitters in organic light-emitting diodes (OLEDs) due to their bright, narrowband emission, which provides a solution to the color saturation required by industry for ultra-high definition (UHD) displays. Here, we report the smallest three boron doped MR-TADF emitter (TBDON), a design that fuses two boron-contacting MR-TADF emitters, DOBNA and ADBNA-Me-Mes, together. The resulting emitter, TBDON, shows desirable narrowband pure blue emission (*λ*_PL_ = 472 nm with FWHM = 28 nm) and efficient TADF with efficient reverse intersystem crossing (RISC), supported by a relatively fast *k*_RISC_ of 7.8 × 10^4^ s^−1^. The OLED with TBDON showed a high maximum external quantum efficiency (EQE_max_) of 24.4%, an EQE of 17.2% at 100 cd m^−2^, and Commission Internationale de l’Éclairage (CIE) coordinates of (0.14, 0.16). The ternary device employing DMAC-DPS as an assistant dopant showed improved performance with a higher EQE_max_ of 28.1% and milder efficiency roll-off with an EQE_100/1000_ of 24.4/17.5%. The high device performance demonstrates the promise of the proposed molecular design.

## Abstract 2

A three-boron doped MR-TADF emitter, TBDON, was developed and shows fast reverse intersystem crossing and bright, blue narrowband emission. The device with TBDON achieved a maximum external quantum efficiency of 28.1% and low efficiency roll-off.

## Graphical abstract

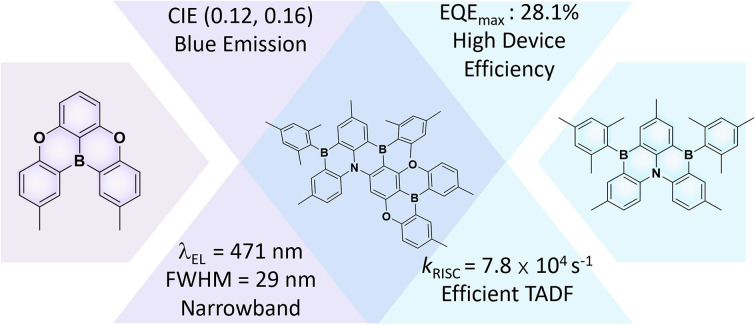

## Introduction

Organic light-emitting diodes (OLEDs) have now taken off as the privileged display technology across a range of consumer electronics categories, including smartwatches, mobile phones, and televisions, and are beginning to appear in an ever increasing number in the automotive sector.^1,2^ However, the external quantum efficiency (EQE) of blue subpixels remains suboptimal as the current materials rely on a triplet–triplet annihilation mechanism for exciton harvesting and thus the internal quantum efficiency is limited to 62.5%.^3^ Multiresonant thermally activated delayed fluorescence (MR-TADF) materials may provide a solution to this outstanding materials design challenge.^4^ These compounds are polycyclic aromatic hydrocarbons (PAHs) typically containing both p- and n-dopants that are judiciously embedded to produce an alternating pattern of increasing and decreasing electron density in the excited state compared to the ground state. This is reflected in the short-range charge transfer (SRCT) character of the lowest lying singlet and triplet excited states that lead to a moderately small singlet–triplet excited-state energy gap (Δ*E*_ST_) that activates reverse intersystem crossing (RISC) channels and turns on TADF.^5,6^ This ingenious molecular design endows MR-TADF emitters with a high photoluminescence quantum yield (*Φ*_PL_), narrowband emission, and 100% exciton utilization efficiency, which make them highly attractive candidates for use in OLEDs.

Since the first examples reported by Hatakeyama and co-workers, numerous blue emissive boron-containing MR-TADF OLED emitters (*e.g.*, DOBNA,^7^ and DABNA,^8^ and ADBNA-Me-Mes^9^) have been reported.^10,11^ However, due to their relatively large Δ*E*_ST_ (∼0.20 eV), most emitters suffer from quite slow RISC (*k*_RISC_ < 10^4^ s^−1^), which leads to undesirably severe efficiency roll-off. Extending the MR-TADF-skeleton to include multi-doped boron/nitrogen atoms can facilitate a more delocalized SRCT excited state, leading to smaller Δ*E*_ST_, thereby accelerating the RISC process.^12–14^ Hatakeyama *et al.* first introduced this extended design strategy in 2018, wherein they altered the number of boron atoms across the emitters B2 and B3 (Fig. S10†). By increasing the number of embedded boron atoms from two to three, Δ*E*_ST_ values for B2 and B3 decreased from 0.19 to 0.15 eV, respectively, in 1 wt% doped films in 3,3′-di(9*H*-carbazol-9-yl)-1,1′-biphenyl (mCBP).^15^ The linear two boron embedded MR-TADF emitter *v*-DABNA (Fig. S10†) was subsequently reported by the same group, which showed a much smaller Δ*E*_ST_ (0.02 eV) and faster *k*_RISC_ (2.0 × 10^5^ s^−1^) in 1 wt% doped films in DOBNA-OAr compared to DABNA-1 (Fig. S10†), with a Δ*E*_ST_ of 0.20 eV and a *k*_RISC_ of 9.9 × 10^3^ s^−1^ in 1 wt% doped films in mCBP.^8,16^ This work directly supports the hypothesis that having an extended π-network is necessary for MR-TADF compounds having a relatively smaller Δ*E*_ST_ and faster *k*_RISC_. There are now numerous examples of two-boron-embedded MR-TADF emitters with small Δ*E*_ST_ < 150 meV and *k*_RISC_ ≥ 10^5^ s^−1^ (*e.g.*, BOBS-Z,^17^BN3,^18^ and TPD4PA^19^). However, examples of MR-TADF emitters incorporating three or more boron atoms into the PAH skeleton are far fewer in number and this is hypothesized to lead to even more efficient RISC. Hatakeyama *et al.* reported a π-extended helical MR-TADF emitter, V-DABNA-Mes,^20^ which has a *k*_RISC_ of 4.4 × 10^5^ s^−1^ in 1 wt% doped films in poly(methylmethacrylate) (PMMA). This translated to an efficient solution-processed OLED emitting at a *λ*_EL_ of 480 nm, with corresponding CIE coordinates of (0.09, 0.21), and showing an EQE_max_ of 22.9%. The same group developed a three-boron-containing MR-TADF emitter ω-DABNA^21^ showing a fast *k*_RISC_ of 1.2 × 10^5^ s^−1^. Our group also reported two three-boron-containing linear MR-TADF heptacene systems, α-3BNOH and α-3BNMes, which show a large Δ*E*_ST_ of 0.28 eV for each, thus resulting in a low *k*_RISC_ < 10^3^ s^−1^ in 1 wt% doped films in poly(methyl)methacrylate (PMMA).^22,23^ Hatakeyama and co-workers subsequently reported a series of extended heptadecacene frameworks with the number of embedded boron atoms increasing from four to eight (for example, CzB4-oPh, Fig. S10†), leading to progressively smaller Δ*E*_ST_ values from 0.04 to 0.03 eV and faster *k*_RISC_ from 6.4 to 65.0 × 10^4^ s^−1^, respectively.^24^

**Fig. 1:**
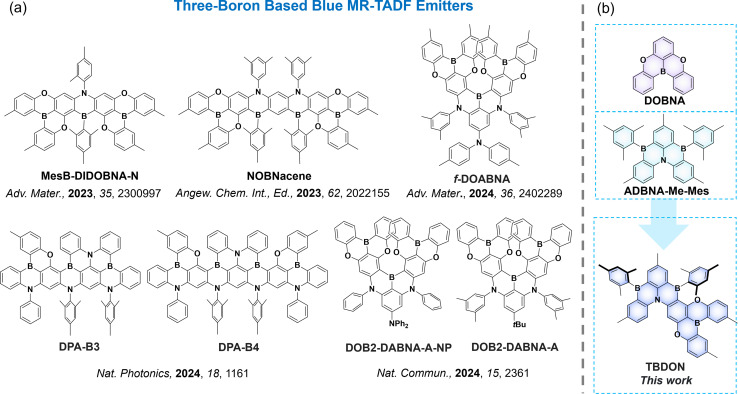
(a) Chemical structures of three boron-based blue MR-TADF compounds and (b) the design strategy of TBDON.

Although these elegant designs demonstrate that *k*_RISC_ can be improved to >10^5^ s^−1^, most of these compounds emit in the sky-blue-to-green region. By constructing twisted-boron-/nitrogen-/oxygen-embedded fused-ring frameworks, Yang *et al.* reported a series of deep blue MR-TADF emitters with the number of boron atoms increasing from two to four, leading to progressively smaller Δ*E*_ST_ values from 0.17 to 0.09 eV and faster *k*_RISC_ from 8 to 30 × 10^4^ s^−1^.^25^ By incorporating the ultraviolet emissive DOBNA motif within a π-extended MR-TADF skeleton, our group has reported a series of deep blue emitters and devices. For instance, MesB-DIDOBNA-N^26^ emits at 402 nm and has a *Φ*_PL_ of 75% but has only a moderate *k*_RISC_ of 9.8 × 10^3^ s^−1^ and large Δ*E*_ST_ of 0.24 eV in a 1.5 wt% doped film in diphenyl[4-(triphenylsilyl)phenyl]phosphine oxide (TSPO1) The OLED with MesB-DIDOBNA-N showed an EQE_max_ of 16.2% and had a CIE_*y*_ coordinate of 0.049. The related four-boron embedded MR-TADF emitter NOBNacene has a slower *k*_RISC_ of 3.7 × 10^3^ s^−1^ as a 1.5 wt% doped film in TSPO1. The OLED with NOBNacene^27^ showed an EQE_max_ of 11.2% at CIE coordinates of (0.18, 0.07). To improve the *k*_RISC_, we developed the V-shaped MR-TADF emitter *f*-DOABNA,^28^ which shows a much faster *k*_RISC_ of 2 × 10^6^ s^−1^ and high *Φ*_PL_ of 90% in 1 wt% doped films in DOBNA-Tol (Fig. S10†). This translated to high performance OLEDs in 1 wt% doped films in 1,3-bis(*N*-carbazolyl)benzene (mCP) with an EQE_max_ of 19.5% and CIE coordinates of (0.15, 0.04); however, efficient roll-off remains problematic with an EQE_100/1000_ of 15.9/7.5%. Using a similar backbone structure, Hatakeyama and co-workers reported the deep blue emitter DOB2-DABNA-A, which shows a similarly fast *k*_RISC_ of 1.1 × 10^6^ s^−1^ and high *Φ*_PL_ of 92% in 1 wt% doped films in PMMA.^29^ The device showed an EQE_max/100/1000_ of 22.8/23.3/21.6% and CIE coordinates of (0.14, 0.05). These examples illustrate that certain multi-boron systems containing the DOBNA skeleton can show significant potential as emitters for deep-blue and stable devices (Fig. 1).

ADBNA-Me-Mes is arguably the smallest MR-TADF emitter containing two boron atoms embedded within a PAH skeleton. This compound shows sky-blue emission peaking at a *λ*_PL_ of 482 nm and a resistance to aggregation due to the presence of the bulky mesityl group.^9^ With the objective of designing an emitter with a faster *k*_RISC_ and showing pure blue emission (CIE_*x*_ + CIE_*y*_ < 0.3), we merged the motifs of ADBNA-Me-Mes with DOBNA into a single molecule, TBDON, a three-boron-containing MR-TADF emitter. In toluene, TBDON exhibits pure blue emissions at a *λ*_PL_ of 462 nm, a value that is intermediate between those of ADBNA-Me-Mes (*λ*_PL_ = 482 nm) and DOBNA (*λ*_PL_ of 398 nm). TBDON has a comparable *Φ*_PL_ of 88%, a shorter delayed lifetime, *τ*_d,avg_, of 35.6 μs, and a narrower emission profile (FWHM of 28 nm) compared to these two reference compounds. Its *k*_RISC_ of 7.8 × 10^4^ s^−1^ in 3 wt% doped films in 2,6-bis[3-(9*H*-carbazol-9-yl)phenyl]pyridine (2,6-DCzPPy) is ten times faster than those of ADBNA-Me-Mes (*Φ*_PL_ = 89%, *τ*_d_ = 165 μs, FWHM = 33 nm and *k*_RISC_ = 7.6 × 10^3^ s^−1^ in 1 wt% doped films in DOBNA-OAr). The device with 4 wt% TBDON in 2,6-DCzPPy showed narrowband blue electroluminescence at a *λ*_EL_ of 471 nm and FWHM of 29 nm, leading to CIE coordinates of (0.12, 0.16). The device showed a relatively mild efficiency roll-off, with an EQE_max/100/1000_ of 24.4/20.2/13.7%. To improve the device performance further, ternary devices containing the assistant dopant 10,10′-(4,4′-sulfonylbis(4,1-phenylene))bis(9,9-dimethyl-9,10-dihydroacridine) (DMAC-DPS) were fabricated, which showed an EQE_max_ of 28.1% and a suppressed efficiency roll-off, with an EQE_100/1000_ of 24.4/17.5%.

## Results and discussion

**Fig. 2:**
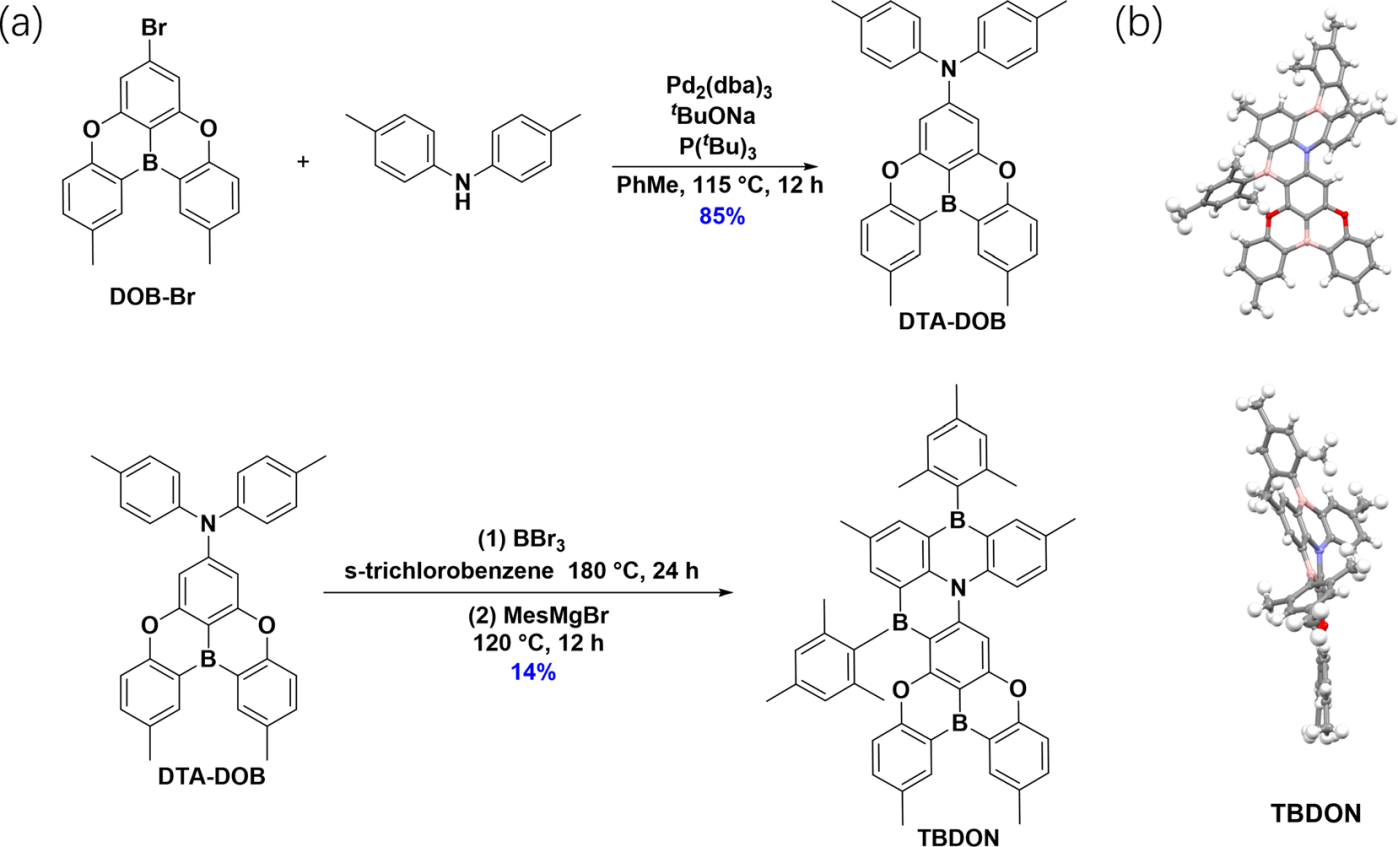
(a) Synthesis route of target TBDON. (b) Single crystal structure of TBDON and its side view. Thermal ellipsoids are displayed at the 50% probability level; solvent is omitted for clarity.

TBDON was synthesized following a two-step linear reaction sequence (Fig. 2a). DOB-Br was synthesized according to a previously reported protocol.^27^DOB-DPA was obtained in a yield of 85% through a palladium-catalyzed Buchwald–Hartwig coupling between DOB-Br and 4,4′-dimethyldiphenylamine. Electrophilic borylation of DOB-DPA with BBr_3_ and quenching with 2-mesitylmagnesium bromide afforded the desired product TBDON in 16% yield; the low yield is attributed to the challenging purification, due to similar *R*_f_ (retention factor) values between TBDON and the impurities; the sample could only be obtained by recrystallization from toluene and ethanol following chromatography. The structure and purity of TBDON were confirmed by ^1^H and ^13^C NMR spectroscopy, high-resolution mass spectrometry (HRMS), single crystal X-ray diffraction (SC XRD) analysis, high performance gel permeation chromatography (GPC), and elemental analysis (Fig. S1–S8†). The thermal stability of TBDON was investigated by thermogravimetric analysis (TGA) (Fig. S15†). This compound is thermally stable, with a 5% mass loss (*T*_d_) occurring at 374 °C.

Single crystals of TBDON (CCDC: 2431387) were obtained by slow diffusion of EtOH vapors into a saturated solution of TBDON in toluene over several days. The crystal structure is shown in Fig. 2b. No intermolecular hydrogen bonding or π–π stacking interactions exist in the crystal structure. TBDON adopts a twisted geometry, with a dihedral angle of 53.48(9)^°^ between the DOBNA unit and an adjacent phenyl ring (Fig. S9†). The two mesityl moieties are oriented approximately orthogonal to the adjacent rings (angles of 73.89(12) and 84.67(12)^°^). All these structural features endow TBDON with a resistance to aggregation in the crystalline state.

**Fig. 3:**
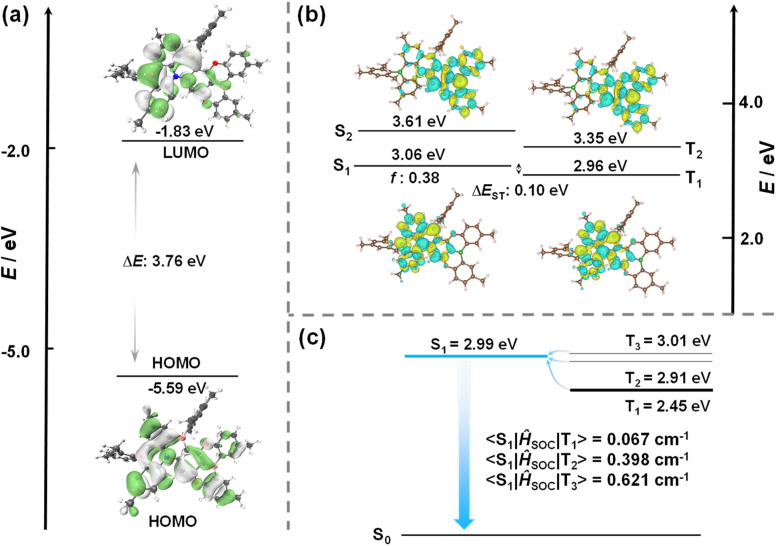
(a) Distributions of the frontier molecular orbitals of TBDON, calculated in the gas phase at the PBE0/6-31G(d,p) level (isovalue: 0.02). (b) Difference density plots of S_1_/S_2_ and T_1_/T_2_ excited states (calculated in the gas phase at the SCS-ADC(2)/cc-pVDZ level) for TBDON (isovalue: 0.02). *f* is the oscillator strength. (c) Spin–orbit coupling matrix element (SOCME) for TBDON based on the optimized T_1_ geometry at the uPBE0/6-31G(d,p) level.

Theoretical calculations were carried out to provide insight into the optoelectronic properties of TBDON. The geometry in the ground state was first optimized using Density Functional Theory at the PBE0/6-31G(d,p) level based on a structure generated from Chem3D, which has a similar geometry to that found in the crystal structure. The HOMO is distributed over the whole molecular skeleton, while the LUMO is mainly localized on the ADBNA-Me-Mes portion of the emitter (Fig. 3a). The calculated HOMO and LUMO levels are −5.59/−1.83 eV, which are essentially the same as those of ADBNA-Me-Mes (−5.61/−1.84 eV, Fig. S13a†). There is a small geometric change in the S_1_ state compared to the ground state, with a RMSD of 0.14 Å (Fig. S11†), which indicates that the emission is likely to be narrowband. Excited-state calculations were performed at the SCS-ADC(2)/cc-pVDZ level, which we have previously shown to accurately predict Δ*E*_ST_ in MR-TADF emitters.^30^ The calculated S_1_/T_1_ energies of TBDON are 3.06/2.96 eV, which are slightly higher than those of ADBNA-Me-Mes at 2.81/2.64 eV, implying that the emission should be blue-shifted in the former. Notably, the Δ*E*_ST_ of 0.10 eV is smaller than the 0.17 eV predicted for ADBNA-Me-Mes, attributed to the more separated FMOs in the former, as shown in Fig. 3a and S13b.† There is an expected alternating pattern of increasing and decreasing electron density in the S_1_, S_2_, T_1_ and T_2_ states compared to the ground state that is indicative that these states have SRCT character (Fig. 3b). The difference density plots of the S_1_ and T_1_ states reveal that the electron density is mainly located on the ADBNA-Me-Mes part of the molecule (Fig. S13b†), which is similar to the spin-density distribution (SSD) of the T_1_ state of TBDON (Fig. S12†), indicating that S_1_ and T_1_ each have similar character to those of ADBNA-Me-Mes. The difference density plots of the S_2_ and T_2_ states are mainly located on the DOBNA fragment. DFT calculations predict a small SOC matrix element (SOCME) of 0.067 cm^−1^ between S_1_ and T_1_ at the optimized T_1_ geometry, which is slightly smaller than that (0.099 cm^−1^) of ADBNA-Me-Mes (Fig. S13c†). While TBDON shows a smaller SOCME between S_1_ and T_1_, the closely lying T_2_ and T_3_ states have a much larger SOC to S_1_ (0.291 and 0.621 cm^−1^, respectively), reflecting the significant differences in orbital types between these excited states (Fig. 3c). These calculations suggest that faster RISC proceeds *via* T_2_/T_3_ to S_1_ for TBDON.^31^

Inferences from the electrochemistry can provide an indication of the energies of the FMOs. As such, the electrochemical properties of TBDON were investigated using cyclic voltammetry (CV) and differential pulse voltammetry (DPV) in deaerated DCM with 0.1 M tetra-*n*-butylammonium hexafluorophosphate as the supporting electrolyte (Fig. S16 and Table S2†). The CV reveals that the oxidation and reduction processes are irreversible. The oxidation and reduction potentials, *E*_ox_ and *E*_red_, determined, respectively, from the first oxidation and reduction peaks of the DPV, are 1.33 and −1.73 V *vs.* the saturated calomel electrode (SCE), while the redox gap, Δ*E*, is 3.06 V. The corresponding HOMO and LUMO energies are −5.67 and −2.61 eV.

**Fig. 4:**
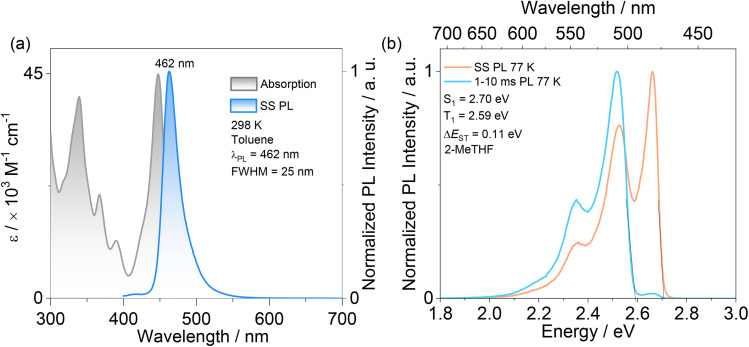
(a) Absorption and steady-state PL (SS PL) spectra in toluene at room temperature (*λ*_exc_ = 340 nm); (b) SS PL and time-gated PL (1–10 ms) emission spectra after Jacobian transformation measured in 2-MeTHF glass at 77 K (*λ*_exc_ = 340 nm).

We next investigated the photophysical properties of the monomolecular species of TBDON in dilute toluene solution (10^−5^ M). The UV/visible absorption spectrum (Fig. 4a) shows intense bands peaking at a *λ*_abs_ of 339 (molar absorptivity, *ε*, of 40.4 × 10^3^ M^−1^ cm^−1^) and 366 nm (20.5 × 10^3^ M^−1^ cm^−1^). The bands ranging from 300 to 380 nm are attributed to π–π^*^ transitions localized over the whole skeleton, assigned from the TD-DFT calculations (Fig. S14†). The lower energy bands peaking at 390 nm are attributed to transitions localized on the DOBNA unit given the similar wavelength to the low energy band of *t*BuDOBNA (*λ*_abs_ = 383 nm).^32^ The intense absorption band peaking at a *λ*_abs_ of 448 nm (*ε* = 44.9 × 10^3^ M^−1^ cm^−1^) corresponds to the SRCT transition associated with the ADBNA unit, which is blue-shifted compared to the SRCT absorption band (*λ*_abs_ = 458 nm) of ADBNA-Me-Mes in DCM.^9^

The photoluminescence (PL) spectrum of TBDON in toluene is narrowband (FWHM of 25 nm), peaking desirably at a *λ*_PL_ of 462 nm. The *λ*_PL_ of TBDON is intermediate to those of ADBNA-Me-Mes (*λ*_PL_ of 482 nm) and DOBNA (*λ*_PL_ of 398 nm) in DCM.^7^ There is also a small Stokes shift of 24 nm (Fig. 4a); this and the narrowband emission reflect the rigid nature of this emitter. There is modest positive solvatochromism of the PL (Fig. S17†), which is consistent with the emissive excited state of SRCT character classifying this compound as an MR-TADF emitter.

The S_1_/T_1_ energies, determined from the onsets of the prompt fluorescence and phosphorescence spectra at 77 K in 2-MeTHF glass, are 2.70 and 2.59 eV (Fig. 4b). The corresponding Δ*E*_ST_, calculated from the difference in energy between these two states, is 0.11 eV, which matches the ADC(2) calculated value of 0.10 eV. The steady-state PL spectra and time-resolved PL decays in aerated toluene show that there is partial quenching of the emission due to O_2_ (Fig. S18†); indeed, the *Φ*_PL_ decreases from 84 to 68% upon exposure to air. The prompt and delayed emission lifetimes, *τ*_p_ and *τ*_d,avg_, in degassed toluene solution are 4.2 ns and 13.2 μs, respectively. Rate constants for the various kinetics processes are summarized in Table S3† and the rate constant for reverse intersystem crossing, *k*_RISC_, is 5.6 × 10^4^ s^−1^. All these results indicate that TBDON in toluene solution emits *via* TADF.

**Fig. 5:**
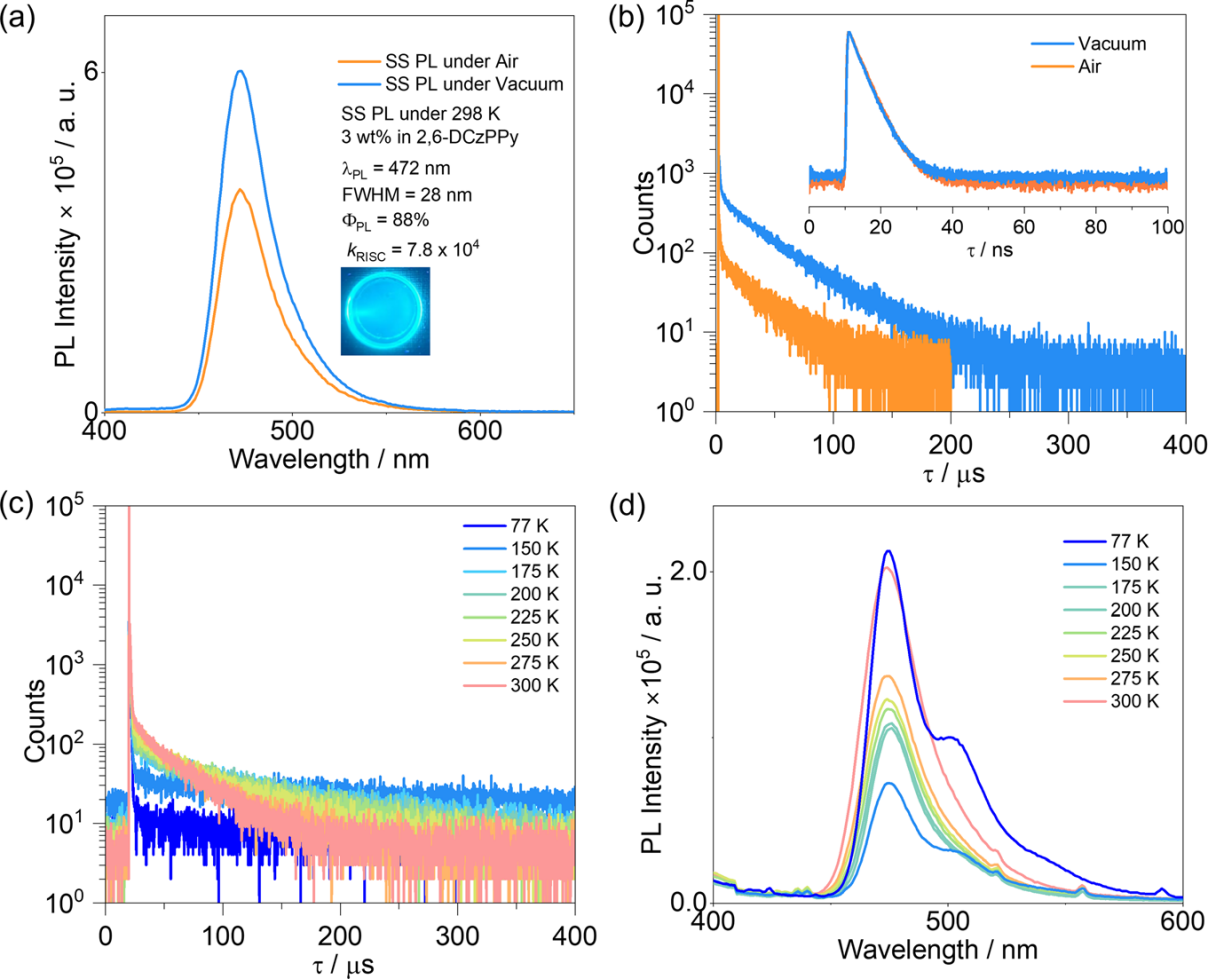
(a) Comparison of the intensity of the PL spectra under vacuum and in air (*λ*_exc_ = 340 nm); (b) time-resolved PL decays (*λ*_exc_ = 379 nm) in 3 wt% 2,6-DCzPPy doped films. Temperature-dependent (c) TR PL decay (*λ*_exc_ = 379 nm) and (d) SS PL spectra (*λ*_exc_ = 340 nm) of 3 wt% TBDON in 2,6-DCzPPy.

With the goal of employing TBDON as an emitter in OLEDs, we next investigated its photophysical properties in doped films. After a doping concentration screening study in mCP (*E*_T_1__ = 2.81 eV),^33^ which identified 3 wt% doping as optimal, and a targeted host screen (Fig. S19 and S20†), we identified 2,6-DCzPPy as the best host. We thus investigated in more detail the photophysics of TBDON in this host (Table 1). In 3 wt% doped films in 2,6-DCzPPy, TBDON shows narrowband blue emission at a *λ*_PL_ of 472 nm (FWHM of 28 nm), which is slightly red-shifted compared to that in toluene. This emission, however, is blue-shifted and more narrowband compared to the emission of ADBNA-Me-Mes in DOBNA-OAr host (*λ*_PL_ = 482 nm; FWHM = 33 nm).^9^ The SS PL intensity in air is lower than that seen under vacuum, indicating that there are likely triplet excitons being quenched. The S_1_/T_1_ energies, determined from the onsets of the SS PL and delayed emission spectra at 77 K (Fig. S21†), are 2.69/2.57 eV, respectively, with a corresponding Δ*E*_ST_ of 0.12 eV. This value is almost identical to the value in 2-MeTHF and smaller than the Δ*E*_ST_ values of ADBNA-Me-Mes (0.18 eV in 1 wt% doped films in DOBNA-OAr) and DOBNA (0.18 eV in 1 wt% doped films in PMMA).^9,34^ The activation energy Δ*E*^TADF^_a_ is estimated to be 60 meV (Fig. S22†). The small activation energy in 2,6-DCzPPy may indicate the involvement of higher-lying triplet excited states in the RISC process. The time-resolved PL (TR-PL) decay shows obvious prompt and delayed components, implicating TADF. Under vacuum, the prompt PL decays with a lifetime, *τ*_p_, of 4.0 ns and the delayed PL decays with a lifetime, *τ*_d,avg_, of 35.6 μs. The prompt lifetime is almost identical to the value in toluene solution, while the *τ*_d,avg_ is much longer, indicating that non-radiative decay is suppressed in the doped film. Notably, the *τ*_d,avg_ of the 3 wt% doped film of TBDON in 2,6-DCzPPy is much shorter than that of the 1 wt% doped film of ADBNA-Me-Mes in DOBNA-OAr (*τ*_d,avg_, of 165 μs), arising from the smaller Δ*E*_ST_ in the former.^9^ The excited-state kinetics were determined based on the measured *Φ*_PL_ and lifetimes (Table S3†). The *k*_RISC_ of 7.8 × 10^4^ s^−1^ for TBDON is over 10 times faster than that of ADBNA-Me-Mes (7.6 × 10^3^ s^−1^), indicating that there is a more efficient upconversion of triplet excitons to singlets in TBDON, benefitting from its shorter *τ*_d,avg_.^12^ Temperature-dependent SS PL and TR PL measurements demonstrate that an increase in the delayed emission is responsible for the increase in intensity of the PL as the temperature increases (Fig. 5c and d).

**Table 1:** Photophysical data of TBDON in doped films in 2,6-DCzPPy

Emitters	*Φ*_PL_ a/%	*λ*_PL_ b/nm	FWHM^c^/nm	S_1_^d^/eV	T_1_^e^/eV	Δ*E*_ST_^f^/eV	*τ*_p_ g/ns	*τ*_d,avg_ h/μs	*k*_RISC_ i/10^4^ s^−1^
TBDON	88	472	28	2.69	2.57	0.12	4.0	35.6	7.8
ADBNA-Me-Mes^9^	89	482	33	—	—	0.18	6.0	165	0.76

a Measured using an integrating sphere under nitrogen (*λ*_exc_ = 340 nm).

b Obtained at 298 K, *λ*_exc_ = 340 nm.

c Full-width at half-maximum.

d Obtained from the onset of the SS PL spectrum at 77 K.

e Obtained from the onset of the delayed emission spectrum (1–10 ms) at 77 K (*λ*_exc_ = 340 nm).

f Δ*E*_ST_ = *E* (S_1_) − *E* (T_1_).

g Measured at 300 K under vacuum by time-correlated single photon counting (TCSPC).

h Measured at 300 K under vacuum by MCS, *λ*_exc_ = 379 nm.

i The calculation methodology is described in the ESI and data are provided in Table S3.^35^

**Fig. 6:**
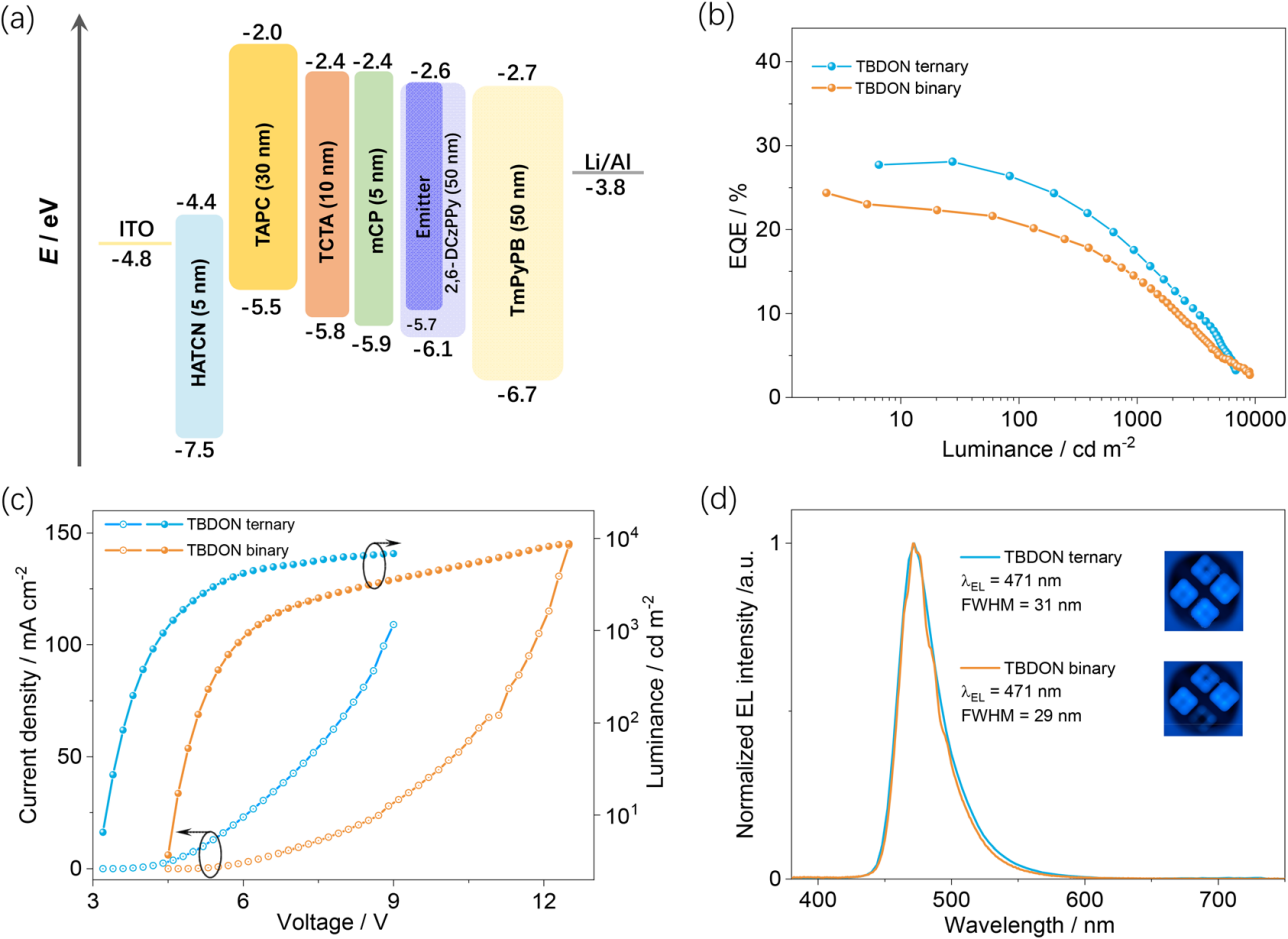
(a) Device configuration and energy levels for each layer; (b) EQE *versus* electroluminescence characteristics. (c) *J*–*V*–*L* characteristics; (d) electroluminescence spectra for devices; inset: images of working devices.

The excellent photophysical performance of TBDON encouraged us to evaluate this compound as an emitter in vacuum-deposited OLEDs. Based on prior work fabricating blue OLEDs we used the following device structure:^32^ ITO/HATCN (5 nm)/TAPC (30 nm)/TCTA (10 nm)/mCP (5 nm)/2,6-DCzPPy:TBDON (*x* wt%) (20 nm)/TmPyPB (40 nm) LiF (1 nm)/Al (100 nm), where indium tin oxide (ITO) is the anode, 1,4,5,8,9,11-hexaazatriphenylenehexacarbonitrile (HATCN) is the hole injection layer, both 4,4′-cyclohexylidenebis[*N*,*N*-bis(4-methylphenyl)benzenamine] (TAPC) and tris(4-carbazoyl-9-ylphenyl)amine (TCTA) act as hole transporting layers, mCP acts as an exciton blocking layer, 1,3,5-tri(*m*-pyridin-3-ylphenyl)benzene (TmPyPB) is the electron transporting material, and LiF modifies the work function of the aluminum cathode. The chemical structures of these materials are shown in Fig. S23,† the device performance is summarized in Fig. 6 and S25† and the data are collated in Table 2 and S4†.

**Table 2:** Data of devices with binary and ternary emissive layers

Device	*V*_on_ a/V	*λ*_EL_/nm	FWHM^b^/nm	CIE	EQE_max/100/1000_^c^/%	*L*_max_ d/cd m^−2^
ADBNA-Me-Mes^9^	—	481	32	(0.10, 0.27)	16.2/11.1/—	<1000
TBDON (binary)	4.3	471	29	(0.12, 0.16)	24.4/20.2/13.7	8800
TBDON (ternary)	3.2	471	30	(0.12, 0.17)	28.1/24.4/17.5	6800

a Turn on voltage, recorded at 1 cd m^−2^.

b Full width at half-maximum of the electroluminescence spectrum.

c Maximum external quantum efficiency/EQE at 100 cd m^−2^/EQE at 1000 cd m^−2^.

d Maximum luminance.

Due to the differing film preparation methods between the photophysical studies and the devices, we fabricated devices using different doping concentrations of TBDON in the emissive layer, ranging from 2 to 5 wt%, (Fig. S25†). All the devices show maximum external quantum efficiencies, EQE_max_, of more than 20%. With increasing emitter doping concentrations from 2 to 5 wt%, the efficiency roll-off decreased, ostensibly due to an improved charge mobility balance. The devices show narrowband blue emission across the range of emitter doping concentrations investigated, with *λ*_EL_ at around 470 nm, and corresponding CIE coordinates of (0.12, 0.15). These devices are blue-shifted compared to those employing ADBNA-Me-Tip and ADBNA-Me-Mes [*λ*_EL_ of 481 and 480 nm and CIE coordinates of (0.10, 0.27) and (0.11, 0.29), respectively].^9^ The highest EQE_max_ of 24.4% was achieved with 4 wt% TBDON, with an associated color point characterized by a *λ*_EL_ of 471 nm, an FWHM of 29 nm and CIE coordinates of (0.12, 0.16). Gratifyingly, compared to the rather severe efficiency roll-off in the devices with ADBNA-based emitters (EQE_max_/EQE_100_ = 16.2/11.1% and 21.4/15.4% for the devices with ADBNA-Me-Tip and ADBNA-Me-Mes, respectively), the devices with TBDON showed milder efficiency roll-off, with an EQE_100_ of 20.2% and EQE_1000_ of 13.7%, respectively, which is attributed to the faster *k*_RISC_ from TBDON.

In an effort to improve the device performance, we opted to explore a ternary emissive layer architecture where an assistant dopant would serve to more efficiently harvest excitons and then transfer these to TBDON, acting as the terminal emitter. Based on an analysis of the spectral overlap between potential TADF emitters and the absorption of TBDON, we identified DMAC-DPS as a suitable assistant dopant capable of efficiently engaging in Förster resonance energy transfer (FRET) with TBDON (Fig. S26†). The faster *k*_RISC_ of 1.8 × 10^5^ s^−1^ from the 10 wt% doped film of DMAC-DPS in mCP^36^ should contribute to reducing the efficiency roll-off in the device. Ternary devices were fabricated by incorporating 20 wt% DMAC-DPS as the assistant dopant into the EML and 2 wt% of TBDON as the terminal emitter (Fig. 6). Employing the system of TBDON: DMAC-DPS: 2,6-DCzPPy = 2/20/78% as the EML produced devices showing an improved EQE_max_ of 28.1% and alleviated efficiency roll-off, with an EQE_100_ and EQE_1000_ of 24.4 and 17.5%, respectively (Fig. 6b). Unfortunately, despite the higher device efficiencies, the efficiency roll-off was not significantly improved. To understand the origin of this surprising result, we explored the photophysical properties of spin-coated films emulating this ternary EML (Fig. S27†). The ternary film has a slightly lower *Φ*_PL_ of 82% (88% for the binary film) and an almost identical SS PL spectrum to the binary film (Fig. S27a†), indicating that there is efficient energy transfer from DPS-DMAC to TBDON. However, the *τ*_d,avg_ of the ternary film remains effectively unchanged, with a slightly shorter *τ*_d,avg_ of 31.5 μs (Fig. S27b†), compared to 35.6 μs for the binary film, leading to a similar *k*_RISC_ of 6.34 × 10^4^ s^−1^ (Table S3†), indicating that TBDON remains engaged in the triplet exciton upconversion process. However, the figure of merit for TADF emitters *k*^S^_r_*K*_eq_ of the ternary film is higher at 3.6 × 10^4^ s^−1^ compared to 3.3 × 10^4^ s^−1^ for the binary film, indicating more efficient exciton harvesting in the ternary system, which explains the lower efficiency roll-off.

## Conclusions

Here, we have developed a tri-boron based MR-TADF emitter (TBDON) by fusing MeDOBNA and ADBNA-Me-Mes together. TBDON emits desirably at an intermediate pure blue emission (*λ*_PL_ of 472 nm) between the sky blue of ADBNA-Me-Mes and the purple of DOBNA. TBDON has a high *Φ*_PL_ of 88%, moderate Δ*E*_ST_ values of 0.12 eV, and shorter delayed lifetimes of 35.6 μs in 3 wt% doped films in 2,6-DCzPPy. OLEDs using this emitter showed efficient performance with an EQE_max_ of 24.4%, relatively mild efficiency roll-off (EQE_100/1000_ = 20.2/13.7%), and narrowband blue emission peaking at 471 nm, with CIE coordinates of (0.12, 0.16). To further optimize the device performance, the D-A TADF emitter, DMAC-DPS, was employed as an assistant dopant in a ternary device configuration. These devices showed a higher EQE_max_ of 28.1%, without adversely affecting the color purity.

## Author contributions

Sen Wu and Dongyang Chen contribute equally to this paper. Sen Wu: conceptualization, investigation, writing – original draft preparation, review & editing. Dongyang Chen: investigation for devices, review & editing. Mathilde Seinfeld: part of synthesis, review & editing. Aidan P. McKay and David B. Cordes: crystal analysis, review & editing. Xiaohong Zhang: resources, review & editing. Eli Zysman-Colman: project management, supervision, resources, writing – review & editing.

## Conflicts of interest

There are no conflicts to declare.

## Supplementary Material

SC-016-D5SC03560K-s001

SC-016-D5SC03560K-s002

SC-016-D5SC03560K-s003

## Data Availability

The research data supporting this publication can be accessed at https://doi.org/10.17630/e2d9724d-fe70-4034-9441-e20db3dfcbf8. The X-ray structural data can be obtained from the Cambridge Crystallographic Data Centre (https://www.ccdc.cam.ac.uk/structures) as deposition number 2431387.
