# Supplementary material for: A three boron doped B/O/N multi-resonant TADF emitter for improved reverse intersystem crossing rate and efficient pure blue organic light-emitting diodes
Source: Chem Sci. 2025 Jul 10;16(33):15256–64. doi: 10.1039/d5sc03560k (PMC12302026; doi:10.1039/d5sc03560k)
Supplement: SC-016-D5SC03560K-s002 [file SC-016-D5SC03560K-s002.pdf]

Supplementary Information (SI) for Chemical Science.
This journal is © The Royal Society of Chemistry 2025

A Three Boron doped B/O/N Multi-Resonant TADF Emitter for Improved Reverse Intersystem Crossing Rate and Efficient Pure Blue Organic Light Emitting Diodes

Sen Wu,[a] *Dongyang Chen,*[a,b] *Mathilde Seinfeld,*[a] *Aidan P. McKay,*[a] *David B. Cordes,*[a] *Xiaohong Zhang*[b,c] and *Eli Zysman-Colman*[a*]

[a]Organic Semiconductor Centre, EaStCHEM School of Chemistry, University of St Andrews, St Andrews, Fife, UK, KY16 9ST, Fax: +44-1334 463808; Tel: +44-1334 463826; E-mail: eli.zysman-colman@st-andrews.ac.uk;

[b]Institute of Functional Nano & Soft Materials (FUNSOM), Joint International Research Laboratory of Carbon-Based Functional Materials and Devices, Soochow University, Suzhou, Jiangsu 215123, P. R. China. Email: xiaohong_zhang@suda.edu.cn

[c]Jiangsu Key Laboratory of Advanced Negative Carbon Technologies, Soochow University, Suzhou, 215123, Jiangsu, P. R. China.

Table of Contents

General methods..**S3**

Experimental method **S**7

Computations **S16**

Optoelectronic Characterization..**S1**9

Devices..**S2**4

References **S28**

General methods

General Synthetic Procedures. The other reagents and solvents were obtained from commercial sources and used as received unless otherwise stated. Air-sensitive reactions were done under a nitrogen atmosphere using Schlenk techniques. Dry solvents used in the reaction were obtained from a MBRAUN SPS5 solvent purification system. Flash column chromatography was carried out using silica gel (Silia-P from Silicycle, 60 Å, 40-63 μm). Analytical thin-layer-chromatography (TLC) was performed with silica plates with aluminium backings (250 μm with F-254 indicator). TLC visualization was accomplished by 254/365 nm UV lamp. HPLC was conducted on a Shimadzu LC-40 HPLC system. HPLC traces were performed using a Shim-pack GIST 3μm C18 reverse phase analytical column. ^{1}H and ^{13}C and NMR spectra were recorded on a Bruker Advance spectrometer (400 MHz for ^{1}H and 101 MHz for ^{13}C). The following abbreviations have been used for multiplicity assignments: “s” for singlet, “d” for doublet, “m” for multiplet, “dd” for doublet of doublets, “ddd” for doublet of doublets of doublets. ^{1}H and ^{13}C NMR spectra were referenced to the solvent peaks). Melting points were measured using open-ended capillaries on an Electrothermal 1101D Mel-Temp apparatus and are uncorrected. High-resolution mass spectrometry (HRMS) was performed at University of Edinburgh Mass Spectrometry Facility. Elemental analyses were performed by Dr. Joe Casillo at the University of Edinburgh.

Quantum chemical calculations. The ground-state optimization was carried out using Density Functional Theory (DFT) level with Gaussian 16[1] using the PBE0[2] functional and the 6-31G(d,p) basis set,[3] starting from a structure drawn and optimized using Chem3D. The excited-state calculations were performed using Time-Dependent DFT within the Tamm-Dancoff approximation (TDA-DFT)[4,5] with the same functional and basis set as for the ground-state geometry optimization in the gas phase. Spin-orbit coupling matrix elements SOCME were calculated based on the optimized excited triplet state geometry. The molecular orbital distributions were visualized with Gaussview 6.0.[6] For the ADC(2) calculations, the ground states was optimized using the ADC(2)-SCS functional and the cc-pVDZ basis set[7,8] in the gas phase based on the geometry calculated by DFT. Vertical transitions to the excited states were performed based on the ground-state optimized structure. The RMSD of ground state and excited singlet state was visualized using VMD program. Calculations were submitted and processed using the Digichem software package (version 6),[9,10] which incorporates a number of publicly available software libraries, including: cclib[11] for parsing of result files,

VMD[12]/Tachyon[13] for 3D rendering, Matplotlib for drawing of graphs,[14] Open Babel[15]/Pybe[16] for file interconversion and PySOC[17] for the calculation of spin-orbit coupling

Electrochemistry measurements. Cyclic Voltammetry (CV) analysis was performed on an Electrochemical Analyzer potentiostat model 620E from CH Instruments at a sweep rate of 100 mV/s. Differential pulse voltammetry (DPV) was conducted with an increment potential of 0.004 V and a pulse amplitude, width, and period of 50 mV, 0.05, and 0.5 s, respectively. Samples were prepared in DCM solutions, which were degassed by sparging with DCM-saturated nitrogen gas for 5 minutes prior to measurements. All measurements were performed using a 0.1 M DCM solution of tetra-*n*-butylammonium hexafluorophosphate, [^{n}Bu$_4$N]PF$_6$. An Ag/Ag$^+$ electrode was used as the reference electrode while a platinum electrode and a platinum wire were used as the working electrode and counter electrode, respectively. The redox potentials are reported relative to a saturated calomel electrode (SCE) with a ferrocenium/ferrocene (Fc/Fc$^+$) redox couple as the internal standard (0.46 V vs SCE).[18] The HOMO and LUMO energies were determined using the relation $E_{HOMO/LUMO} = -(E_{ox} / E_{red} + 4.8)$ eV,[19] where E_{ox} and E_{red} are the onset of anodic and cathodic peak potentials, respectively calculated from DPV relative to Fc/Fc$^+$.

Photophysical measurements. Optically dilute solutions of concentrations on the order of 10^{-5} or 10^{-6} M were prepared in spectroscopic grade solvents for absorption and emission analysis. Absorption spectra were recorded at room temperature on a Shimadzu UV-2600 double beam spectrophotometer with a 1 cm quartz cuvette. Molar absorptivity determination was verified by linear regression analysis of values obtained from at least four independent solutions at varying concentrations range from 3.0×10^{-6} to 1.0×10^{-5} with absorbance ranging from 0.025 to 0.100. For emission studies, steady-state emission and time-resolved emission spectra were recorded at room temperature using an Edinburgh Instruments FS5 fluorimeter. Samples were excited at 340 nm for steady-state measurements and 379 for time-resolved PL decays. Photoluminescence quantum yields for solutions were determined using the optically dilute method, in which four sample solutions with absorbances of ca. 0.10, 0.075, 0.050 and 0.025 at 360 nm were used. The Beer-Lambert law was found to remain linear at the concentrations of the solutions. For each sample, linearity between absorption and emission intensity was verified through linear regression analysis with the Pearson regression factor (R^2) for the linear fit of the data set surpassing 0.9. Individual relative quantum yield values were calculated for each solution and the values reported represent the slope obtained from the linear fit of these

results. The quantum yield of the sample, Φ_{PL}, was determined using the equation $\Phi_{PL} = (\Phi_r * \frac{A_r}{A_s} * \frac{I_s}{I_r} * \frac{n_s^2}{n_r^2})$, where A stands for the absorbance at the excitation wavelength (λ_{exc} = 340 nm), I is the integrated area under the corrected emission curve and n is the refractive index of the solvent with the subscripts "s" and "r" representing sample and reference respectively.[20] Φ_r is the absolute quantum yield of the external reference quinine sulfate (Φ_r = 54.6% in 1 *N* H_2SO_4).[21]

An integrating sphere (Edinburgh Instruments FS5, SC30 module) was employed for the photoluminescence quantum yield measurements of thin film samples. The Φ_{PL} of the films were then measured in air and in N_2 by purging the integrating sphere with N_2 gas flow for 2 min. The photophysical properties of the film samples were measured using an Edinburgh Instruments FS5 fluorimeter. Time-resolved PL measurements of the thin films were carried out using the multi-channel scaling (MCS) and time-correlated single-photon counting (TCSPC) technique. The samples were excited at 379 nm by a pulsed laser and were kept in a vacuum of $< 8 \times 10^{-4}$ mbar. The singlet and triplet state energies in 2-MeTHF glass and in doped film were determined from the onset values of the steady-state photoluminescence PL (SSPL) and phosphorescence spectra at 77 K. The singlet-triplet energy gap (ΔE_{ST}) was estimated from the difference in energy of the steady-state PL and phosphorescence spectra. The samples were excited by a xenon flashlamp emitting at 340 nm (EI FS5, SC-70). Phosphorescence spectra were measured with a time-gated window of 1-10 ms.

Fitting of time-resolved luminescence measurements: Time-resolved PL measurements were fitted to a sum of exponentials decay model, with chi-squared (χ^2) values between 1 and 2, using the EI FS5 Each component of the decay is assigned with a weight, (w_i), which is the contribution of the emission from each component to the total emission.

The average lifetime was then calculated using the following expressions:

1. Two exponential decay model:

$$\tau_{AVG} = \tau_1 w_1 + \tau_2 w_2 \qquad \text{(S1)}$$

with weights defined as $w_1 = \frac{A1\tau_1}{A1\tau_1 + A2\tau_2}$ and $w_2 = \frac{A2\tau_2}{A1\tau_1 + A2\tau_2}$ where A_1 and A_2 are the preexponential-factors of each component.

2. Three exponential decay model:

$$\tau_{AVG} = \tau_1 w_1 + \tau_2 w_2 + \tau_3 w_3 \qquad (S2)$$

with weights defined as $w_1 = \frac{A1\tau_1}{A1\tau_1 + A2\tau_2 + A3\tau_3}$, $w_2 = \frac{A2\tau_2}{A1\tau_1 + A2\tau_2 + A3\tau_3}$ and $w_3 = \frac{A3\tau_3}{A1\tau_1 + A2\tau_2 + A3\tau_3}$ where A_1, A_2 and A_3 are the preexponential-factors of each component.

X-ray structure analysis

X-ray diffraction data for **TBDON** were collected at 100 K using a Rigaku MM-007HF High Brilliance RA generator/confocal optics with XtaLAB P200 diffractometer [Cu Kα radiation (λ = 1.54187 Å)]. Data were collected (using a calculated strategy) and processed (including correction for Lorentz, polarization and absorption) using CrysAlisPro.[22] Structures were solved by dual-space methods (SHELXT[23]) and refined by full-matrix least-squares against F^2 (SHELXL-2019/3[24]). Non-hydrogen atoms were refined anisotropically, and hydrogen atoms were refined using a riding model. All calculations were performed using the Olex2[25] interface. Selected crystallographic data: $C_{59}H_{54}B_3NO_2$, M = 841.46, monoclinic, a = 17.4318(3), b = 7.66062(14), c = 34.8958(4) Å, β = 98.1330(13)°, U = 4613.40(13) Å^3, T = 100 K, space group $P2_1/c$ (no. 14), Z = 4, 38931 reflections measured, 9215 unique (R_{int} = 0.0660), which were used in all calculations. The final R_1 [$I > 2\sigma(I)$] was 0.0818 and wR_2 (all data) was 0.2076. CCDC 2431387 contains the supplementary crystallographic data for this paper. These data can be obtained free of charge from The Cambridge Crystallographic Data Centre via www.ccdc.cam.ac.uk/structures.

OLED Fabrication and Characterization:

OLEDs were fabricated on the indium-tin oxide (ITO) coated transparent glass substrates with multiple layers. The ITO glass substrates have a thickness of ca. 100 nm and a sheet resistance of ca. 30 Ω and were cleaned with optical detergent, deionized water, acetone, and isopropanol successively and then dried in an oven. For vacuum-evaporated OLEDs, the ITO substrates were exposed to UV ozone for 15 minutes initially. All the organic materials were thermally evaporated at a rate of 1 Å s^{-1} under a vacuum of ca. 10^{-5} Torr. Finally, LiF and Al were successively deposited at a rate of 0.1 Å s^{-1} and 5 Å s^{-1}, respectively. Four identical OLED devices were formed on each of the substrates with an emission area of 0.09 cm^2 for each device. The EL performances of the devices were measured with a PHOTO RESEARCH Spectra Scan PR 655 PHOTOMETER and a KEITHLEY 2400 Source Meter constant current source at room temperature.

Experimental methods

2,12-Dimethyl-N,N-di-p-tolyl-5,9-dioxa-13b-boranaphtho[3,2,1-de]anthracen-7-amine (DTA-DOB)

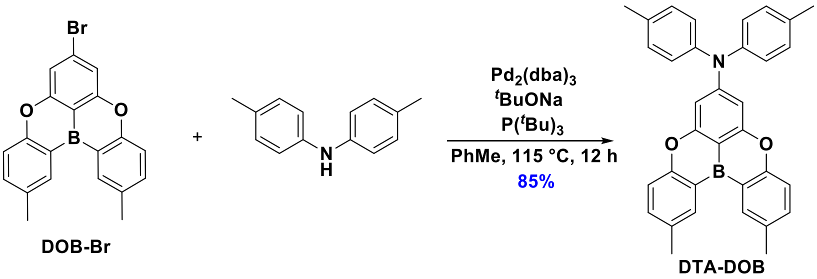

DOB-Br (500 mg, 1.32 mmol, 1 equiv.), di-*p*-tolylamine (314 mg, 1.59 mmol, 1.2 equiv.), $Pd_2(dba)_3$ (61 mg, 0.07 mmol, 0.05 equiv.), tri-*tert*-butylphosphonium tetrafluoroborate (38 mg, 0.13 mmol, 0.1 equiv.), sodium *tert*-butoxide (255 mg, 2.65 mmol, 2.0 equiv.) and 10 mL dry toluene were added to a 50 mL Schlenk tube. After 12 h at 80 °C, the reaction was cooled to RT. The reaction mixture was extracted with DCM (300 mL). The organic layer was then washed with DI water (150 mL × 3). The organic phase was dried over anhydrous sodium sulfate and filtered. The crude product was purified by chromatography on silica gel. Initial elution was (hexane: DCM= 7:3). The corresponding fractions were concentrated under reduced pressure, sonicated for 10 min and filtered. The product was obtained as white solid after filtration and methanol wash (200 mL). **Yield:** 85% (556 mg). **R_f:** 0.30 (hexane: DCM= 7:3) **Mp:** 364-365 ºC. **^{1}H NMR (400 MHz, CD_2Cl_2)** δ 8.48 – 8.41 (m, 2H), 7.48 (dd, J = 8.5, 2.2 Hz, 2H), 7.31 (d, J = 8.5 Hz, 2H), 7.27 – 7.16 (m, 8H), 6.62 (s, 2H), 2.55 (s, 6H), 2.41 (s, 6H). ^{13}C NMR (101 MHz, **CD_2Cl_2**) δ 158.66, 158.48, 154.60, 143.90, 135.05, 134.04, 133.98, 131.82, 130.26, 126.70, 117.65, 98.38, 20.79, 20.71. **HR-MS[M+H]$^+$** Calculated: 494.2286 ($C_{34}H_{28}B_1N_1O_2$); Found: 494.2284.

15,19-dimesityl-3,6,13,17-tetramethyl-15,19-dihydro-9,20-dioxa-10b-aza-4b,15,19-tribo-radinaphtho[3,2,1-de:1',2',3'-qr]pentacene (TBDON)

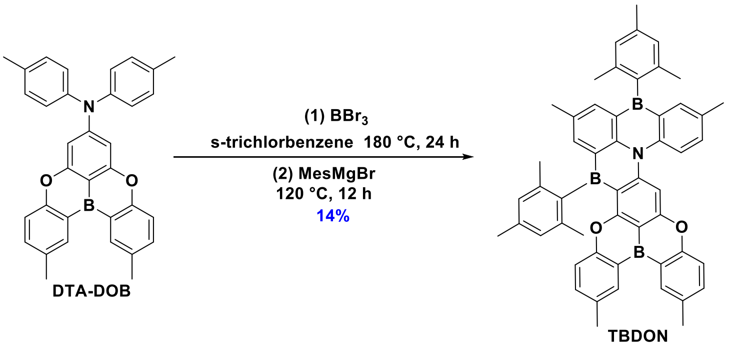

To a solution of **DTA-DOB** (1.10 g, 2.23 mmol, 1.00 equiv.) in 1,2,4-trichlorbenzene (20 mL) was added BBr_3 (2 mL, 21.0 mmol, 9.5 equiv.) dropwise at RT under a nitrogen atmosphere. After stirring at 180 °C for 22 h, excess BBr_3 was distilled off under a positive flow of nitrogen at 180 ºC over 1.5 h. The reaction mixture was then cooled to -78 °C and to this was added dropwise 2-mesitylmagnesium bromide (1 M in THF, 20.00 mL, 20.00 mmol, 8.96 equiv.). After stirring at RT for 2 h, the temperature was increased to 70 °C for 12 h. Upon cooling to room temperature, the reaction mixture was diluted with 100 mL of DCM. The mixture was then transferred to a separating funnel and washed with DI water (3 × 150 mL). The organic phase was dried over anhydrous sodium sulfate, filtered, and reduced under reduced pressure. The crude product was dry-loaded onto silica gel. Initial elution was (hexane: DCM= 4：1). The obtained solid was further purified by slow recrystallization from a Toluene: EtOH mixture. The product was obtained as a yellow solid after filtration. **Yield:** 14% (234 mg). **R_f :** 0.2 (hexane: DCM= 4：1) **Mp:** decomposed at 363-364 ºC. **^{1}H NMR (400 MHz, CD_2Cl_2)** δ 8.54 – 8.46 (m, 2H), 8.30 (d, J = 8.6 Hz, 1H), 8.05 – 8.02 (m, 1H), 7.94 – 7.91 (m, 1H), 7.91 (d, J = 0.5 Hz, 1H), 7.66 (d, J = 2.0 Hz, 1H), 7.55 (dd, J = 8.7, 2.0 Hz, 1H), 7.50 (dd, J = 9.1, 2.3 Hz, 1H), 7.47 – 7.40 (m, 2H), 7.11 (s, 1H), 7.07 (d, J = 5.6 Hz, 2H), 7.00 (s, 1H), 6.37 (d, J = 8.5 Hz, 1H), 2.58 (s, 3H), 2.56 (s, 6H), 2.47 (s, 3H), 2.46 (s, 3H), 2.41 (s, 3H), 2.15 (s, 3H), 2.13 (s, 3H), 2.10 (s, 3H), 2.07 (s, 3H). **^{13}C NMR (101 MHz, CD_2Cl_2)** δ 159.71, 158.49, 152.69, 144.82, 143.37, 143.03, 138.71, 137.77, 136.80, 135.74, 135.25, 134.65, 134.58, 134.42, 133.37, 133.32, 132.70, 132.54, 132.41, 130.85, 127.00, 126.97, 126.79, 126.64, 123.38, 118.36, 117.78, 102.93, 22.97, 22.93, 22.81, 22.66, 21.08, 21.04, 20.88, 20.81, 20.56, 20.49. **HR-MS[M+H]$^+$** Calculated: ($C_{52}H_{46}B_3NO_2$) 750.3880; Found: 750.3882. **Anal. Calcd. For** $C_{52}H_{46}B_3NO_2$: C: 83.35, H: 6.19, N: 1.87. **Found**: C: 83.77, H: 6.18, N: 1.92. 99.02% pure on HPLC analysis, retention time 10.749 minutes in 100% THF.

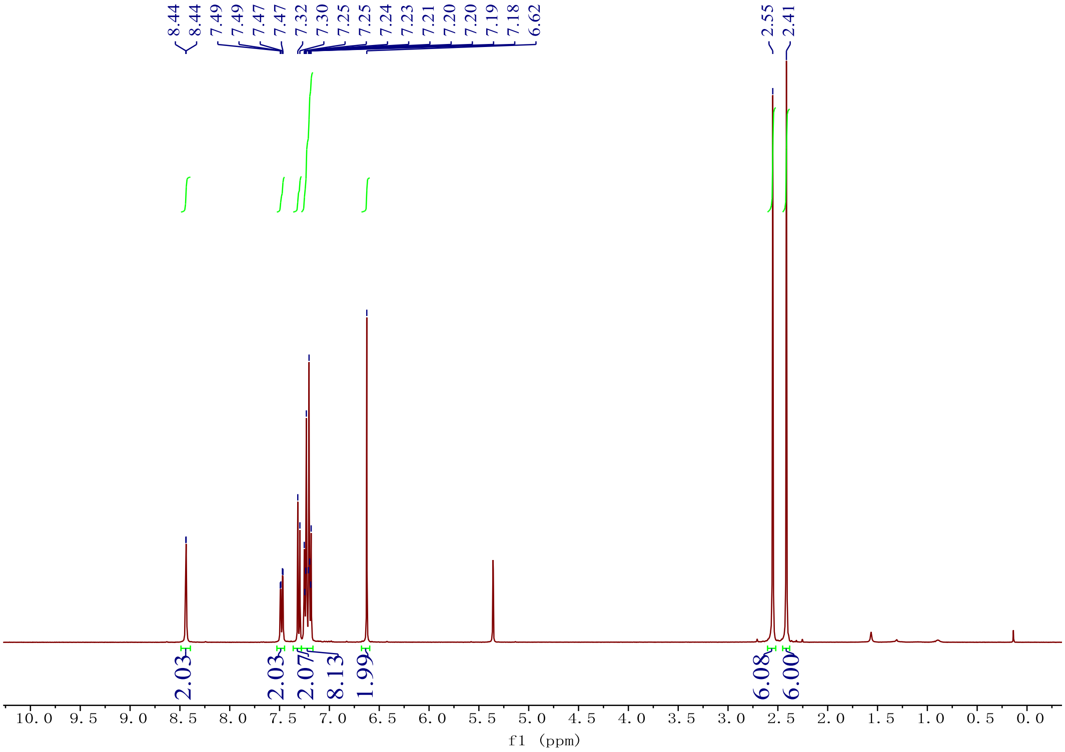

Figure **S1**. ^{1}H-NMR spectrum of **DTA-DOB** in CD_2Cl_2.

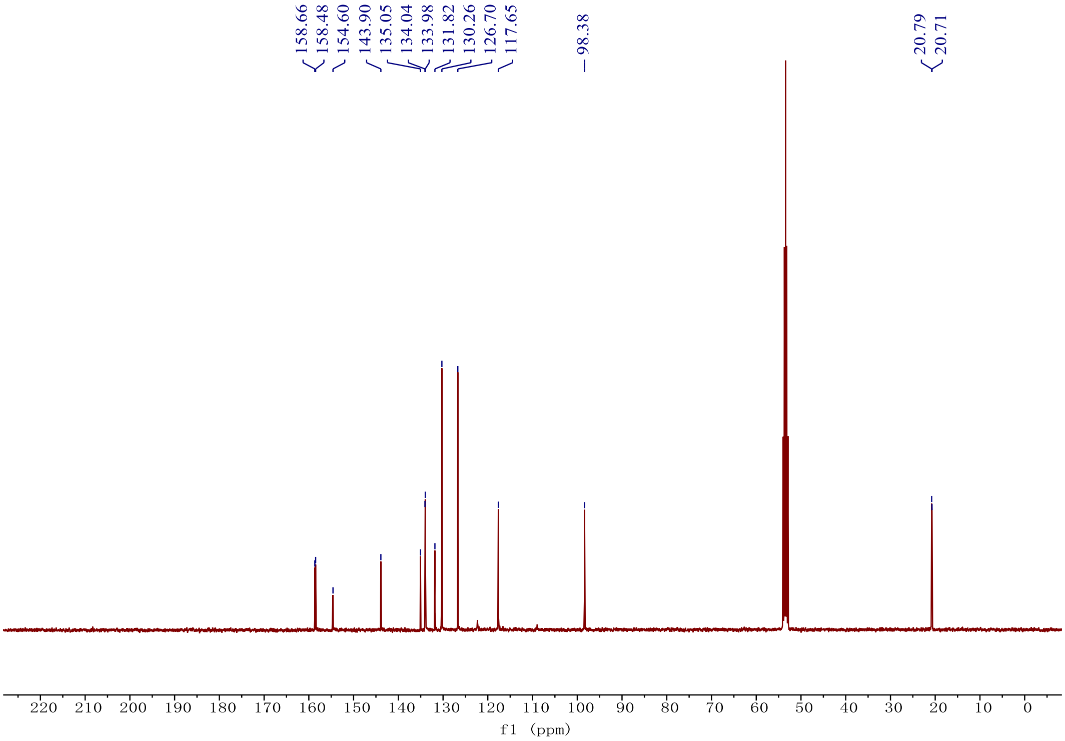

Figure **S2**. ^{13}C-NMR spectrum of **DTA-DOB** in CD_2Cl_2.

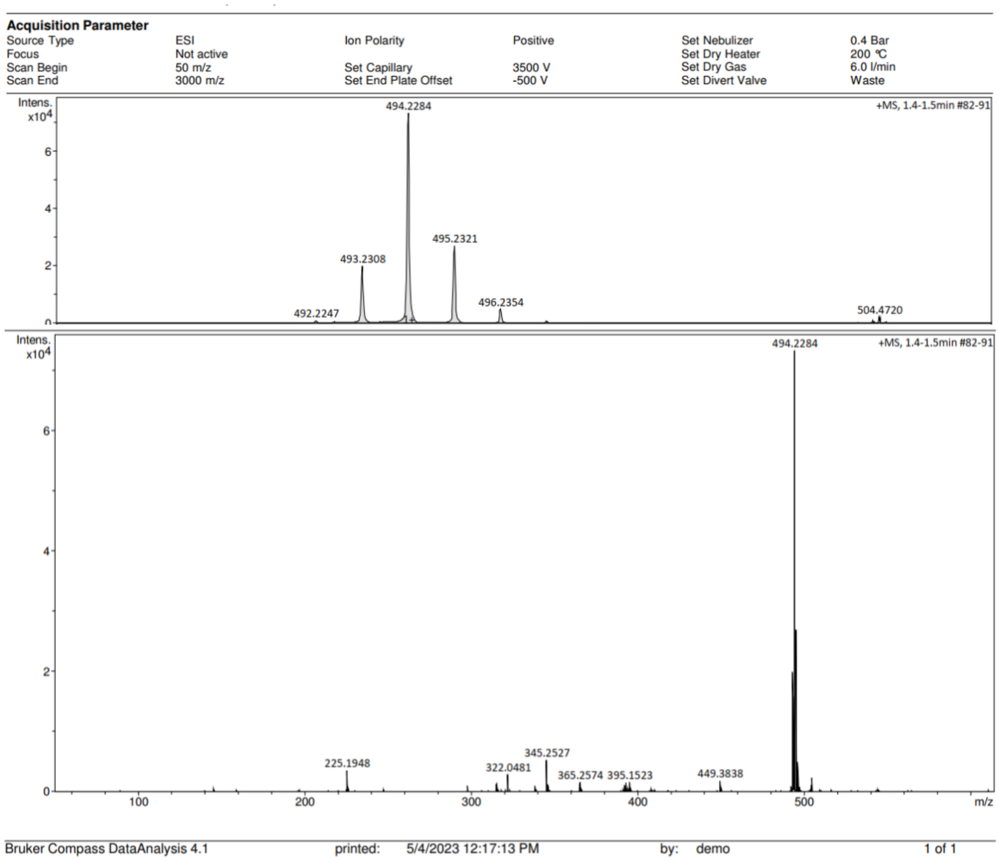

Figure **S3**. ESI-HRMS of **DTA-DOB**.

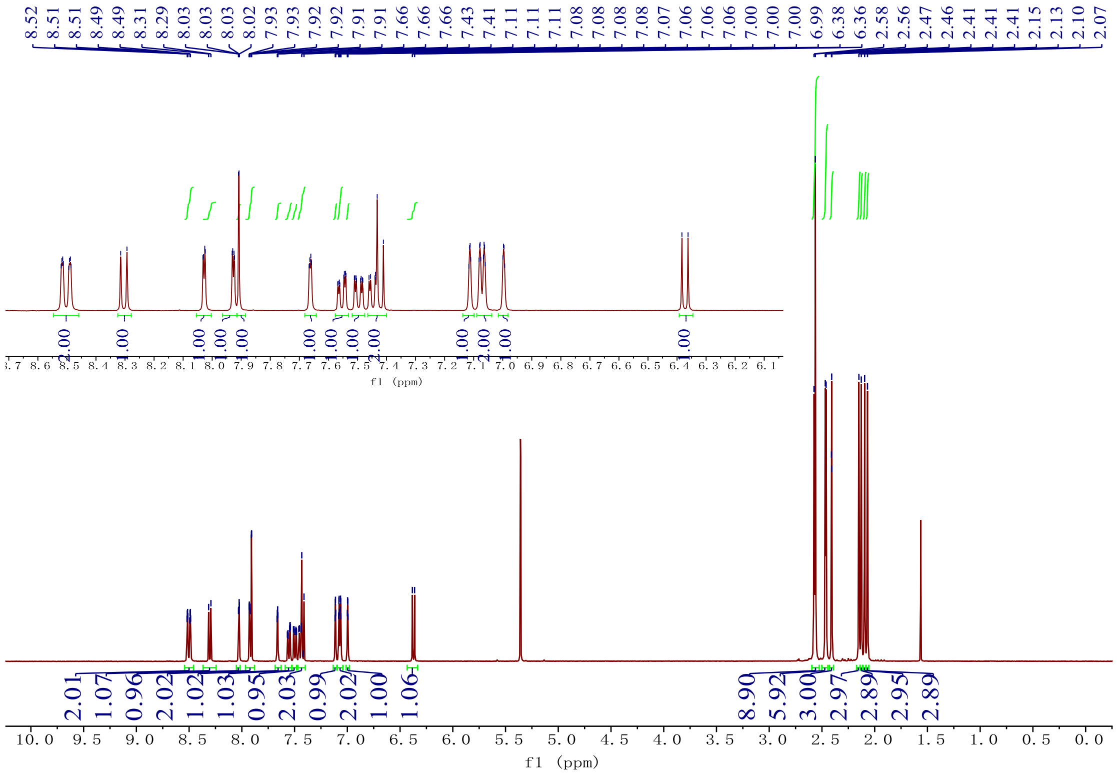

Figure **S4**. ^{1}H-NMR spectrum of **TBDON** in CD_2Cl_2.

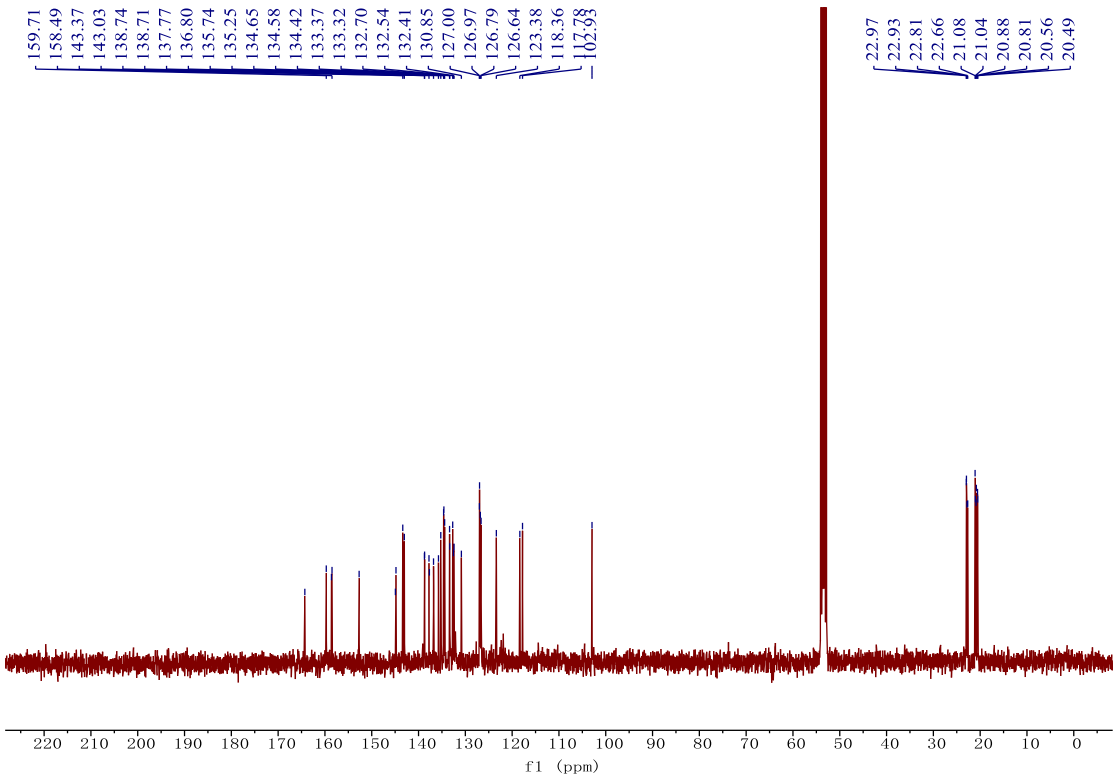

Figure **S5**. ^{13}C-NMR spectrum of **TBDON** in CD_2Cl_2.

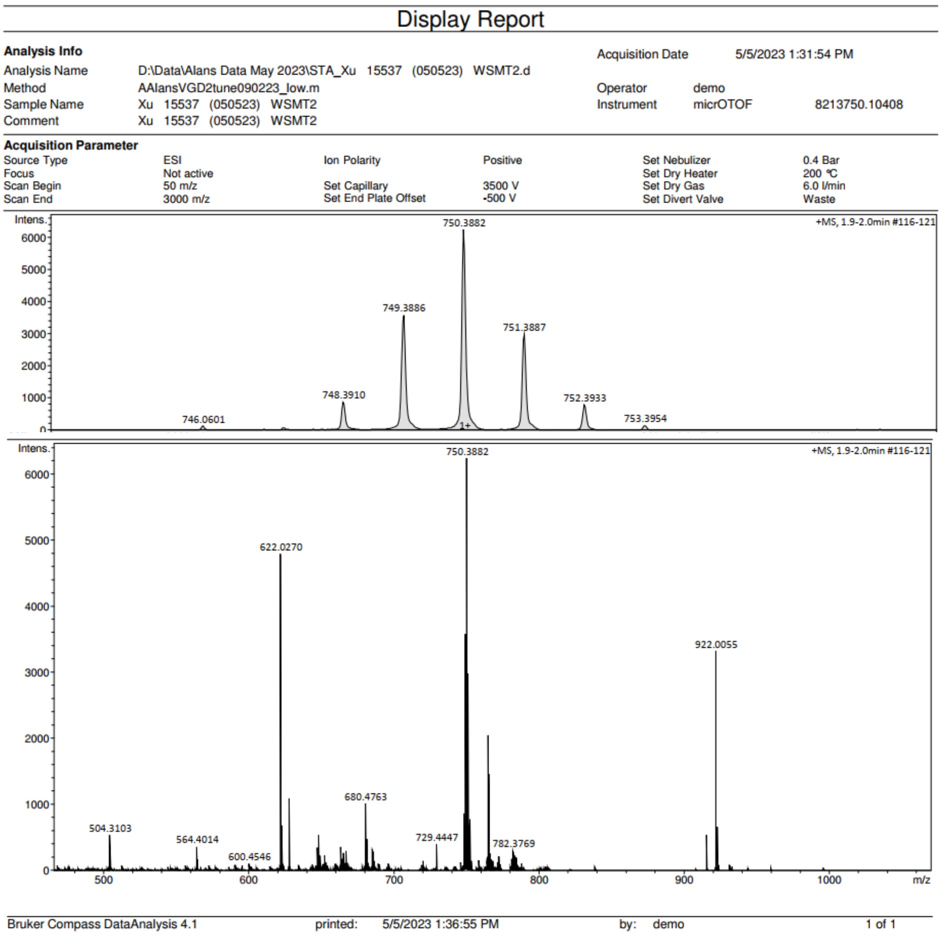

Figure **S6**. ESI-HRMS of **TBDON**.

Elemental Analysis Service Request Form

Researcher name Sen Wu

Researcher email ws60@st-andrews.ac.uk

NOTE: Please submit ca. 10 mg of sample

**Sample reference number**	WS-4
**Name of Compound**	TBDON
**Molecular formula**	C52H46B3NO2
**Stability**	
**Hazards**	
**Other Remarks**	

Analysis type:

Single ☐ **Duplicate** ☐ **Triplicate** ☐

Analysis Result:

Element	Expected %	Found (1)	Found (2)	Found (3)
**Carbon**	83.35	83.70	83.85	
**Hydrogen**	6.19	6.17	6.19	
**Nitrogen**	1.87	1.92	1.92	
**Oxygen**				

Authorising Signature:

**Date completed**	11.10.23
**Signature**	J –PL
**comments**	

Figure **S7**. Elemental Analysis of **TBDON**.

30/05/2022 17:02:55 Page 1 / 1

HPLC Trace Report30May2022

<Sample Information>

Sample Name	: MS7-postRC_2		
Sample ID	: MS7-postRC_2		
Method Filename	: 100% THF 20 mins 280nm - new-please use.lcm		
Batch Filename	: MS7postRC2.lcb		
Vial #	: 1-9	Sample Type	: Unknown
Injection Volume	: 10 uL		
Date Acquired	: 30/05/2022 16:27:19	Acquired by	: System Administrator
Date Processed	: 30/05/2022 16:47:24	Processed by	: System Administrator

<Chromatogram>

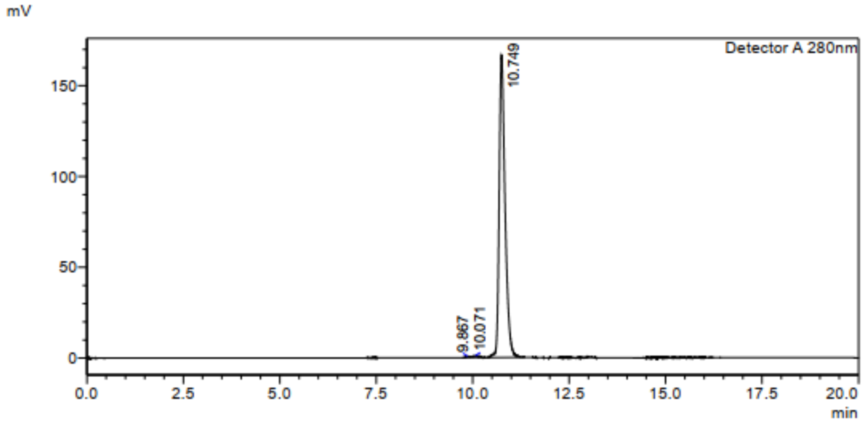

<Peak Table>

Detector A 280nm

Peak#	Ret. Time	Area	Height	Area%	Area/Height	Width at 5% Height
1	9.867	7005	752	0.392	9.313	--
2	10.071	10451	1029	0.584	10.152	--
3	10.749	1771094	166092	99.024	10.663	0.377
Total		1788550	167874	100.000		

Figure **S8**. HPLC analysis of **TBDON**.

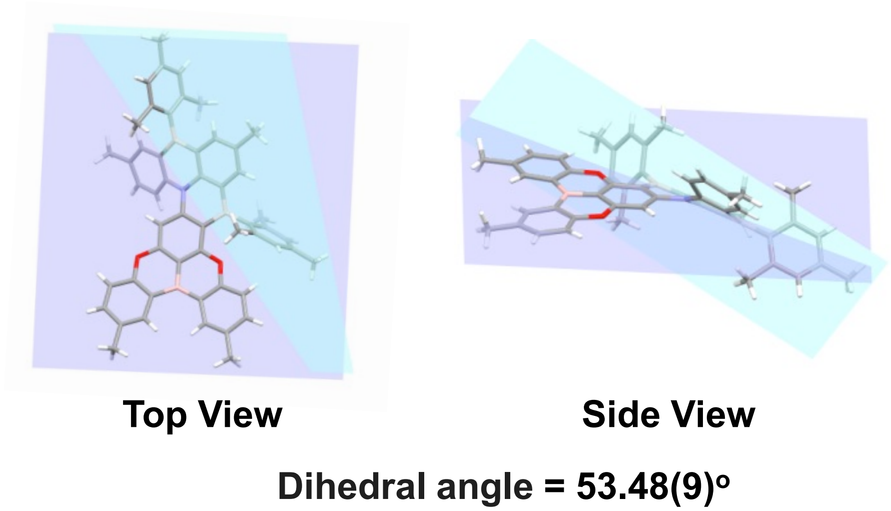

Top View **Side View**

Dihedral angle = 53.48(9)°

Figure **S9**. Dihedral angle between **DOBNA** unite (purple) and adjacent phenyl ring (blue).

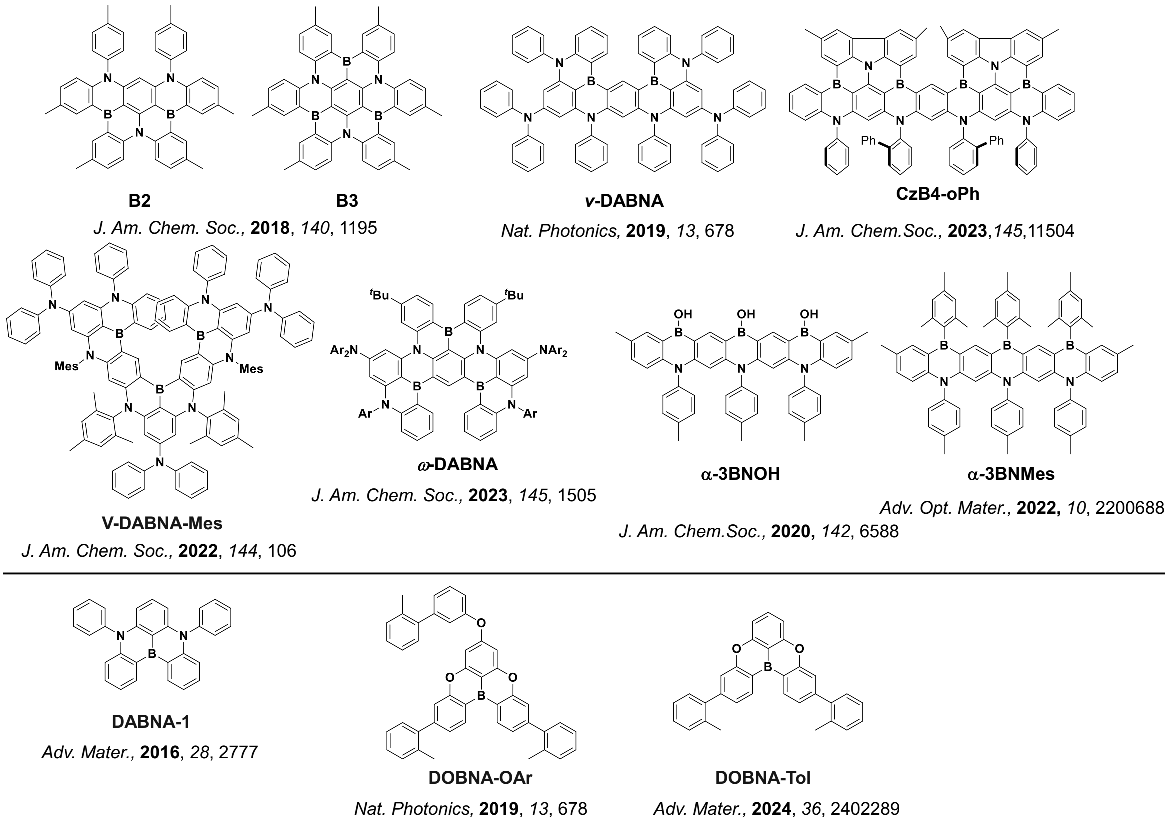

Figure **S10**. Chemical structures of some multi-boron-based MR-TADF compounds and the structures mentioned in the introduction.

Computations

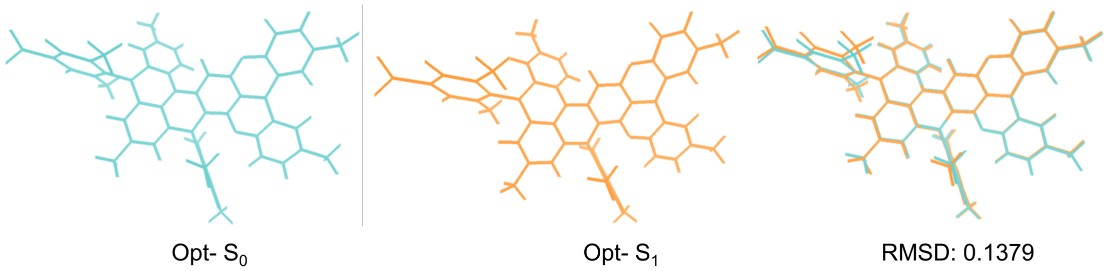

Figure **S11**. Optimized structure of the singlet and ground state, the geometric difference between the S_0 and S_1 states. The root mean square deviation (RMSD) value between the two configurations is 0.1379.

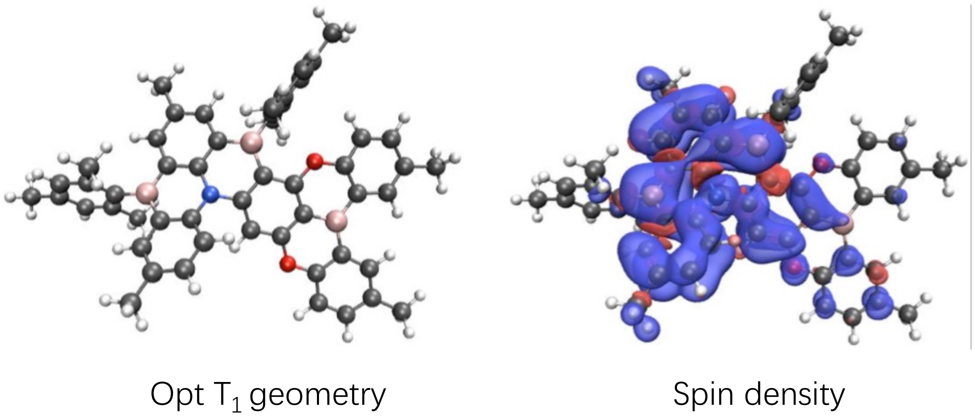

Figure **S12**. Triplet spin density of **TBDON** calculated in the gas phase at the T_1 optimized geometry at the uPBE0/6-31G(d,p) level.

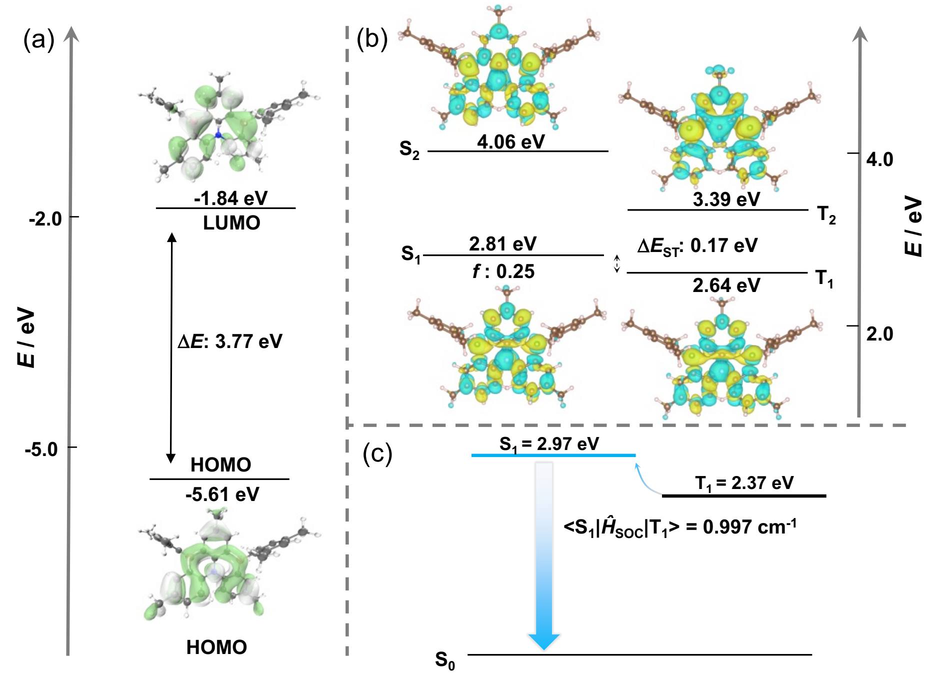

Figure **S13**. a) Distributions of the frontier molecular orbitals of **ADBNA-Me-Mes**, calculated at the PBE0/6-31G(d,p) level in the gas phase (isovalue: 0.02). b) Difference density plots of the S_1/S_2 and T_1/T_2 excited states (calculated at the SCS-ADC(2)/cc-pVDZ level in the gas phase) for **ADBNA-Me-Mes** (isovalue: 0.02). *f* is the oscillator strength. (c) Spin-orbit coupling matrix element (SOCME) for **ADBNA-Me-Mes** based on the optimized T_1 geometry at the uPBE0/6-31G(d,p) level.

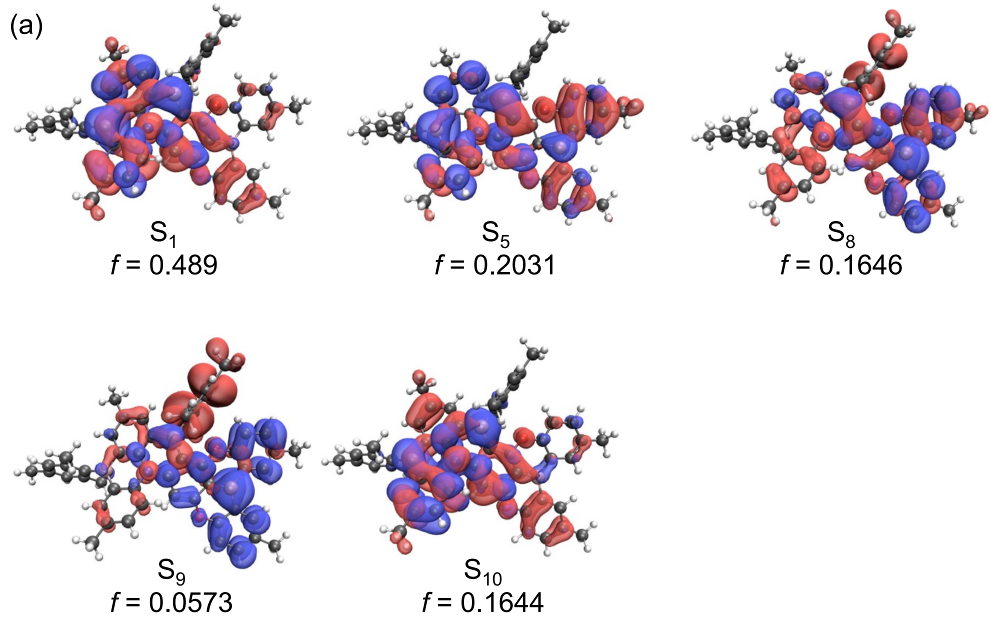

Figure **S14**. The natural transition orbitals (NTOs) for **TBDON** based on the optimized S_0 geometry calculated at the PBE0/6-31G(d,p) level in the gas phase (isovalue: 0.02).

Table **S1**. Table of Excited States and its transitions probability for the singlet excited states, calculated at the SCS-ADC(2)/cc-pVDZ level in the gas phase.

**Compounds**	S_1	S_2
**ADBNA-Me-Mes** (Energy/ eV and transition possibility)	**2.82**<br>HOMO → LUMO (0.92)<br>HOMO-13 → LUMO (0.01)<br>HOMO-12 → LUMO (0.01)<br>HOMO-6 → LUMO+1 (0.01)<br>HOMO-6 → LUMO+3 (0.01)	**4.06**<br>HOMO-5 → LUMO (0.81)<br>HOMO-8 → LUMO (0.02)<br>HOMO → LUMO+2 (0.02)<br>HOMO → LUMO+4 (0.02)<br>HOMO-4 → LUMO (0.02)
**TBDON** (Energy/ eV and transition possibility)	**3.06**<br>HOMO → LUMO (0.82)<br>HOMO → LUMO+1 (0.03)<br>HOMO-1 → LUMO+1 (0.02)<br>HOMO-1 → LUMO (0.01)<br>HOMO-17 → LUMO (0.01)	**3.61**<br>HOMO → LUMO+1 (0.47)<br>HOMO-1 → LUMO+1 (0.26)<br>HOMO-1 → LUMO (0.06)<br>HOMO-8 → LUMO (0.02)<br>HOMO → LUMO+3 (0.01)

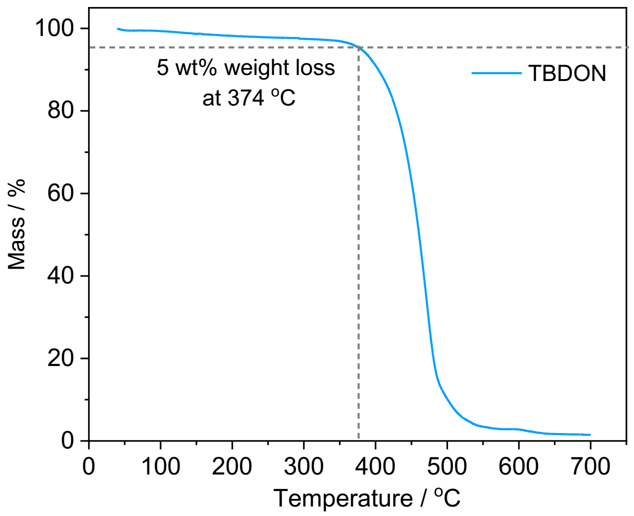

Figure **S15**. TGA analysis of **TBDON** under a N_2 atmosphere (heating rate: 5 °C/ min. The dashed line represents the threshold for a 5 wt% weight loss).

Optoelectronic Characterization

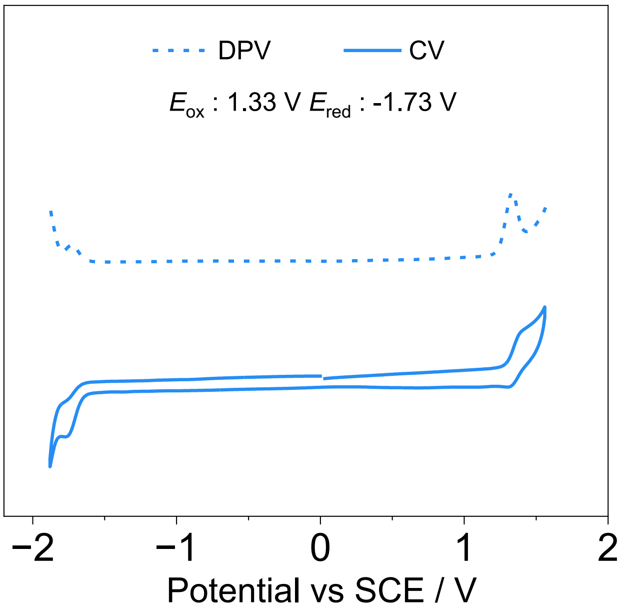

Figure **S16**. Cyclic voltammogram (CV) and differential pulse voltammetry (DPV) in degassed DCM with 0.1 M [^{n}Bu$_4$N]PF$_6$ as the supporting electrolyte and Fc/Fc$^+$ as the internal reference versus SCE (0.46 V vs. DCM)[18].

Table **S2.** Electrochemical data

**Material**	E_{ox} /V^a	E_{red} /V^a	ΔE /V^b	E_{HOMO} / eV^c	E_{LUMO} /eV^c
**TBDON**	1.33	-1.73	3.06	-5.67	-2.61

^{a}E_{ox} and E_{red} are the peak of anodic and cathodic potentials from DPV versus SCE. In degassed DCM with 0.1 M [^{n}Bu$_4$N]PF$_6$ as the supporting electrolyte and Fc/Fc$^+$ as the internal reference (0.46 V vs. SCE)[18]. ^{b}$\Delta E = E_{ox}$-E_{red}. $E_{HOMO/LUMO}$ = -(E_{ox} / E_{red} vs. Fc/Fc$^+$ + 4.8) eV.[19]

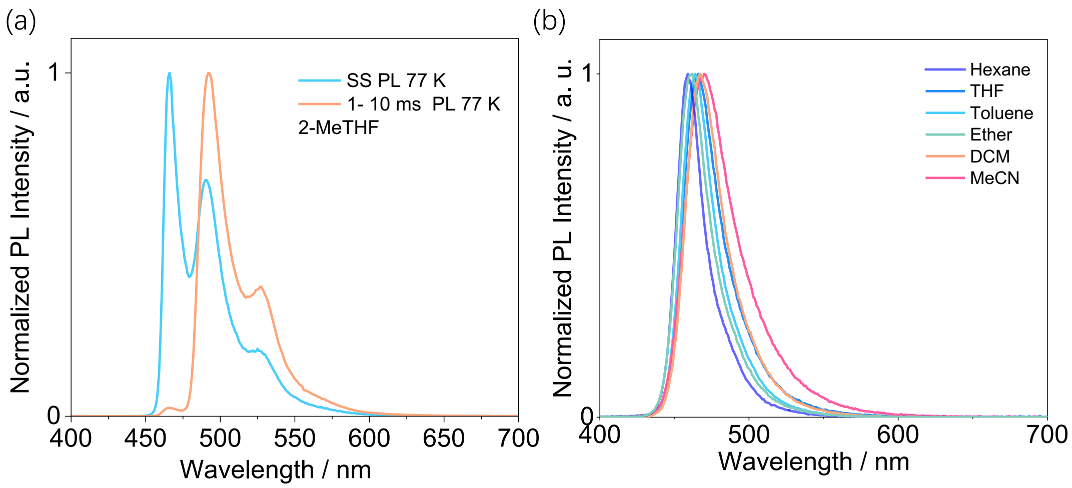

Figure **S17**. (a) Steady-state PL and time-gated PL (1-10 ms) spectra measured in 2-MeTHF glass at 77 K, λ_{exc} = 340 nm. (b) Solvatochromism PL study for **TBDON**. λ_{exc} = 340 nm.

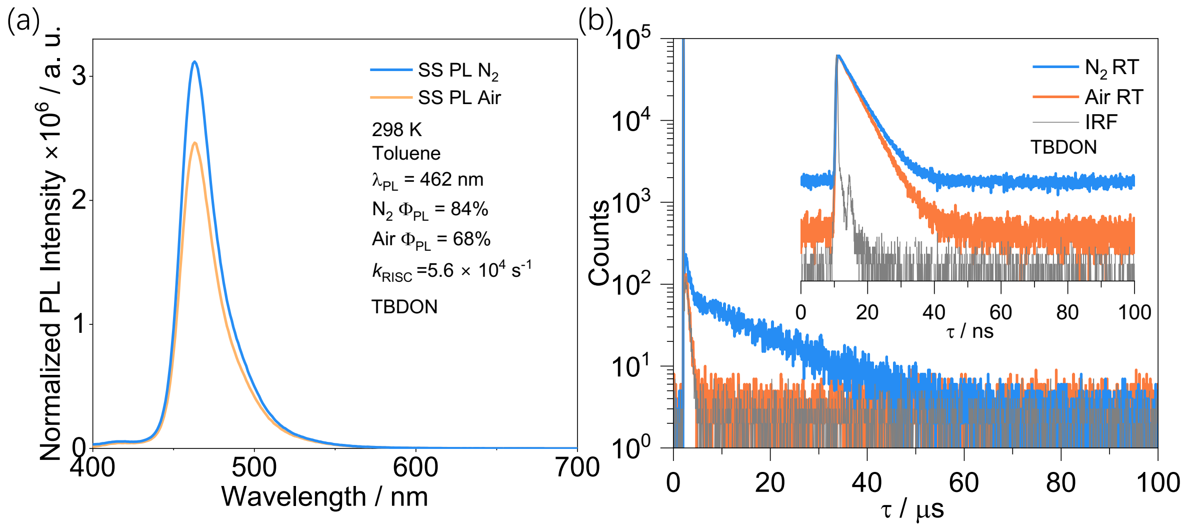

Figure **S18**. (a) Comparison of the intensity of the PL spectra under N_2 and air, λ_{exc} = 340 nm, and (b) Time-resolved PL decay in toluene solution of **TBDON**, λ_{exc} = 375 nm.

With a goal of employing **TBDON** as an emitter in an OLED, we next investigated its photophysical properties in doped films. We started with mCP as a host as it has a suitably high triplet energy (E_{T1} = 2.81 eV)[26] to confine the excitons onto the guest. Firstly, the optimal doping concentration was determined by evaluating the Φ_{PL} as a function of different doping concentrations, ranging from 1.5 to 10 wt% (Figure **S19**). The Φ_{PL} varied narrowly between 78 and 88%, with the highest value observed at 3 wt% doping. We next measured the photophysics of 3 wt% doped films of **TBDON** in two other OLED-relevant hosts 2,8-bis(diphenyl-phosphoryl)-dibenzo[b,d]thiophene (PPT) and 2,6-DCzPPy (Figure **S20**). The SS PL spectra of the 3 wt% doped films in mCP and 2,6-DCzPPy show almost identical profiles,

while the spectrum in PPT is slightly broader. The Φ_{PL} of the 3 wt% doped films in 2,6-DCzPPy of 86% is effectively the same as that in mCP and higher than that of 72% in PPT.

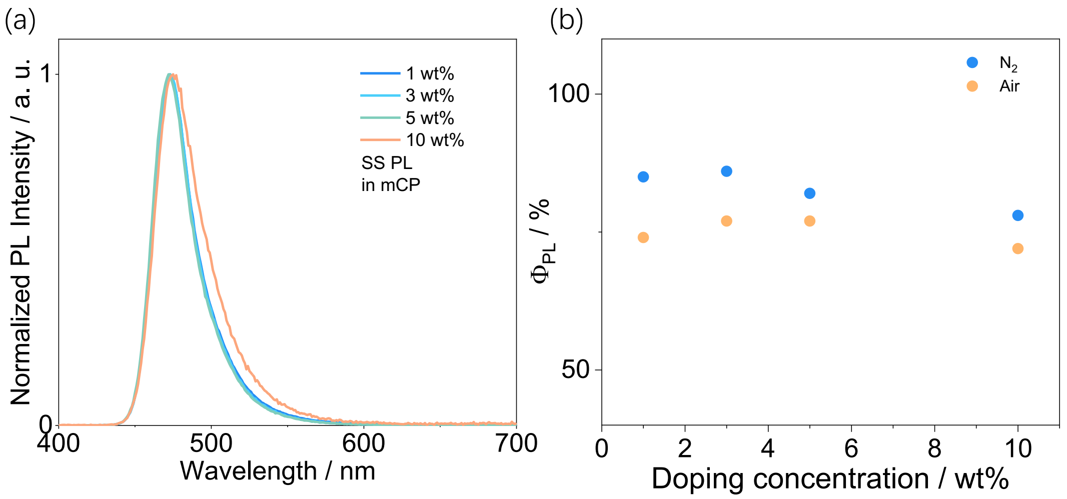

Figure **S19**. (a) Concentration-dependent PL of **TBDON**; (b) Concentration-dependent Φ_{PL} of **TBDON** in mCP doped film under air and nitrogen atmosphere, λ_{exc}=340 nm.

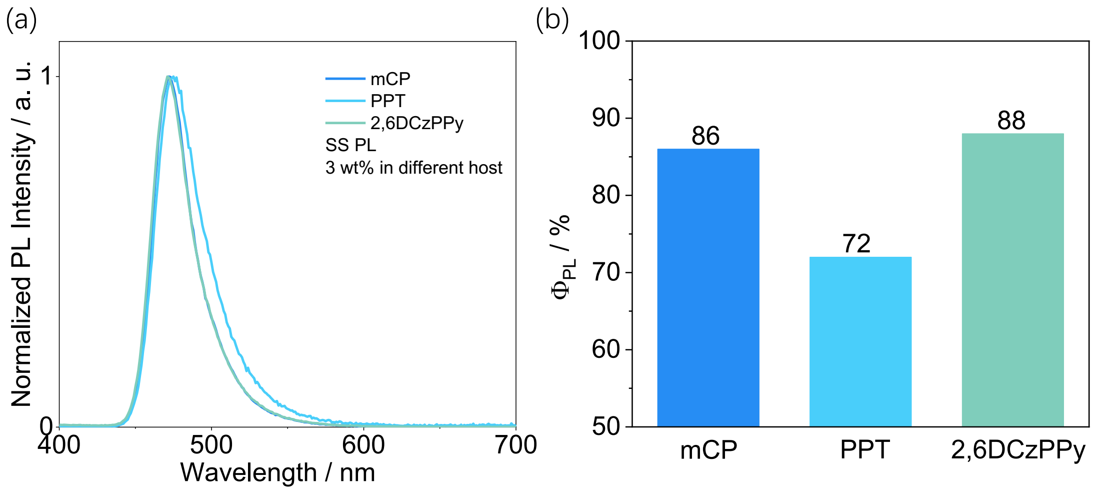

Figure **S20**. (a) SS PL in different host systems of **TBDON**; (b) Φ_{PL} of **TBDON** in different host system under nitrogen atmosphere, λ_{exc}=340 nm.

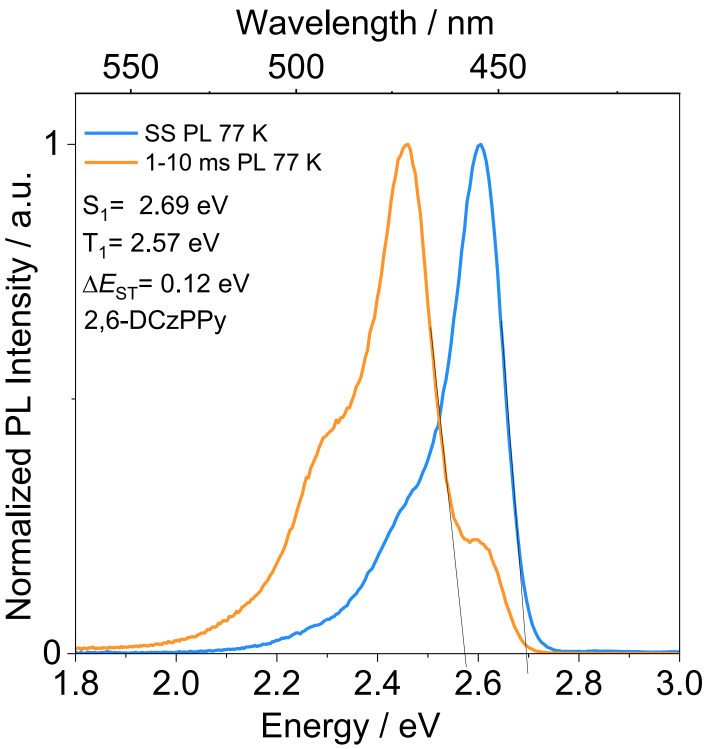

Figure **S21**. Steady-state PL and time-gated PL (1-10 ms) spectra after Jacobian transformation measured as 3 wt% doped film of **TBDON** in 2,6-DCzPPy film, λ_{exc} = 340 nm.

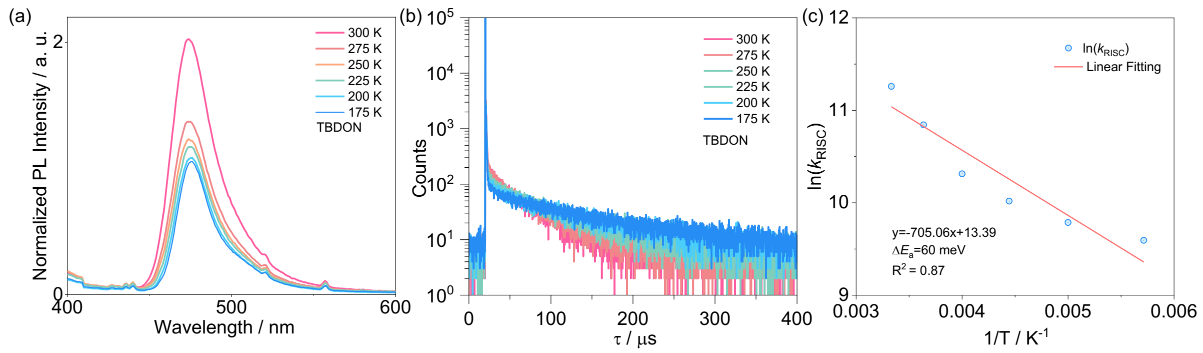

Figure **S22**. Temperature-dependent (a) SS PL spectrum, (b) time-resolved PL decay and (c) Arrhenius plot of 3 wt% doped film of **TBDON** in 2,6-DCzPPy, λ_{exc} = 379 nm.

For a TADF system, the main exciton loss channels are either singlet or triplet nonradiative transition processes. Owing to high performance, the singlet nonradiative transition process (k_{nr}^{S}) can be ignored, therefore the exciton loss can be attributed to the triplet nonradiative transition process (k_{nr}^{T}). Regarding the ternary film system, although the TADF sensitizer **DMAC-DPS** was incorporated into the doped film, the process of RISC in **DMAC-DPS** followed by FRET to **TBDON** appears to be highly inefficient, due to the almost identical delayed lifetimes observed for the ternary (31.5 μs) and binary (35.6 μs) films. Therefore, we

have treated **DMAC-DPS** and 2,6-DCzPPy collectively as the co-host matrix. The kinetics parameters were calculated according to our previous reported paper.[27,28]

Table **S3.** Summary of kinetics parameters of **TBDON** in doped film and solution.

**Matrix**	**T / K**	**Φ_P / %**	**Φ_d / %**	**k_p / 10^7 s^{-1}**	**k_d / 10^3 s^{-1}**	**k_r^S / 10^7 s^{-1}**	**k_{nr}^T / 10^3 s^{-1}**	**k_{ISC} / 10^8 s^{-1}**	**k_{RISC} / 10^4 s^{-1}**	**$k_r^S K_{eq}$ / 10^4 s^{-1}**
toluene	298	68	16	2.38	7.58	16.20	37.9	0.76	5.57	11.8
binary	298	30	58	2.50	2.81	7.50	4.82	1.75	7.76	3.32
	275	28	32	2.50	2.54	7.00	14.00	1.80	4.08	-
	250	28	26	2.50	1.69	7.00	10.90	1.80	2.14	-
	225	26	24	2.50	1.35	6.50	9.14	1.85	1.67	-
	200	27	17	2.50	1.22	6.75	9.33	1.83	1.04	-
	175	29	13	2.50	1.05	7.25	8.59	1.78	0.66	-
ternary	298	36	46	1.52	3.17	5.45	8.93	0.97	6.34	3.56

Binary film: 4 wt% doped film of **TBDON** in 2,6-DCzPPy film; Ternary film: 2 wt% **TBDON**/ 20 wt% **DMAC-DPS** doped in 2,6-DCzPPy film.

Devices

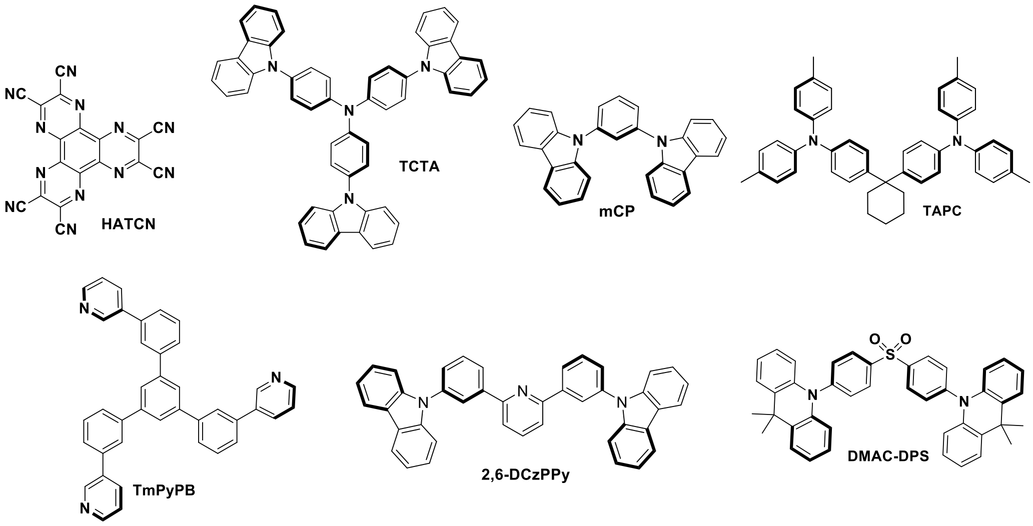

Figure **S23**. Chemical structures of the materials used in the devices.

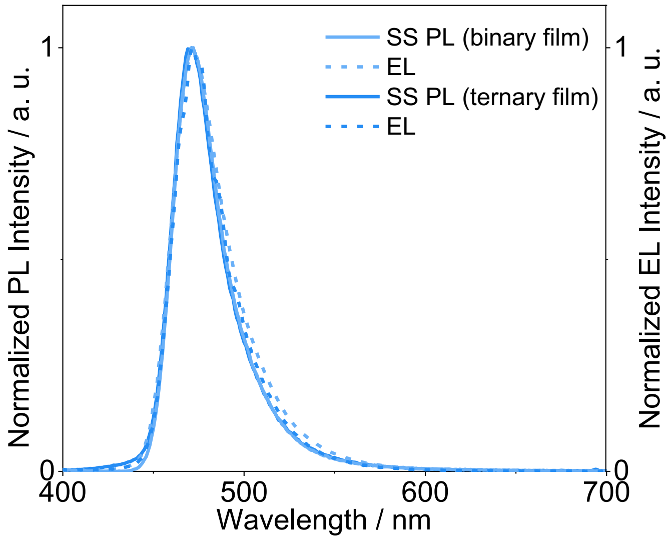

Figure **S24**. Comparation between the steady-state PL and EL spectra, λ_{exc} = 340 nm.

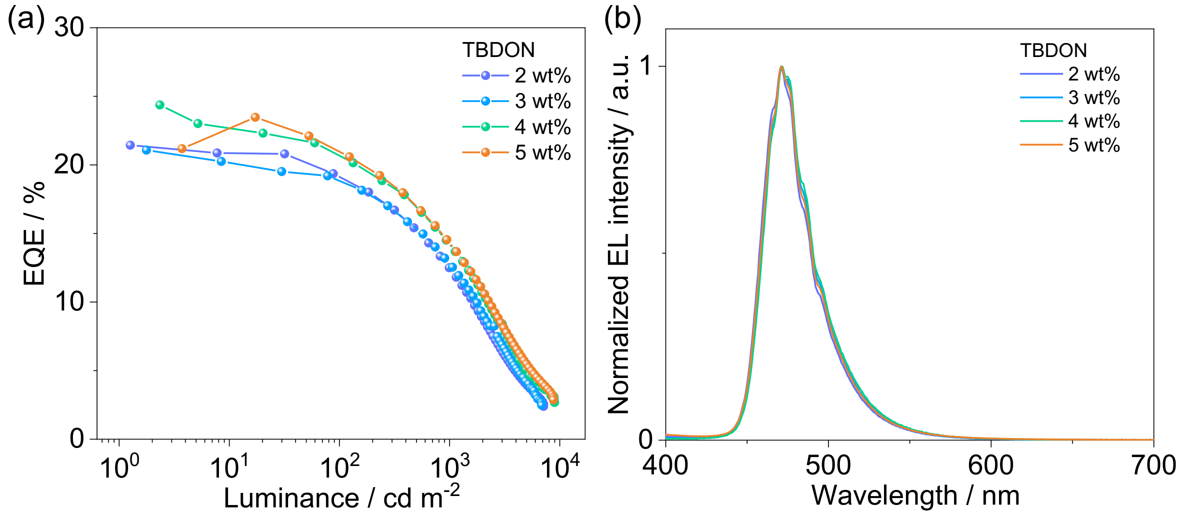

Figure **S25**. (a) EQE versus luminance characteristics and (b) the electroluminescent spectra for devices using different doping concentrations of **TBDON** in 2,6-DCzPPy.

Table **S4**. Electroluminescence data.

**Device (x wt%)**	**V_{on}[a] / V**	**λ_{EL} / nm**	**FWHM[b] / nm**	**CIE (x,y)**	**L_{max}[c] /cd m^{-2}**	**$EQE_{max/100/1000}$[d] / %**
**2**	4.5	470	30	0.12, 0.15	7142	21.4/18.5/12.5
**3**	4.5	470	30	0.12, 0.15	6855	21.1/19.0/12.5
**4**	4.4	470	29	0.12, 0.16	8987	24.4/20.2/13.7
**5**	4.4	471	29	0.12, 0.16	8765	24.4/20.2/13.7

[a]Turn-on voltage at the luminance of 1 cd m^{-2}. [b]Full-width at half-maximum of the EL spectrum. [c]Maximum luminance. [d]EQE_{max}/EQE at 100 cd m^{-2}/EQE at 1000 cd m^{-2}.

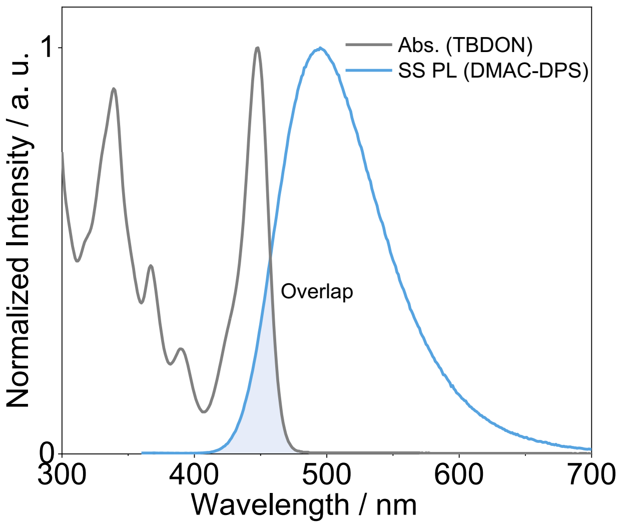

Figure **S26**. The absorption spectrum of **TBDON** in toluene and the SS-PL spectrum of **DMAC-DPS** in 2,6-DCzPPy (λ_{exc} = 340 nm).

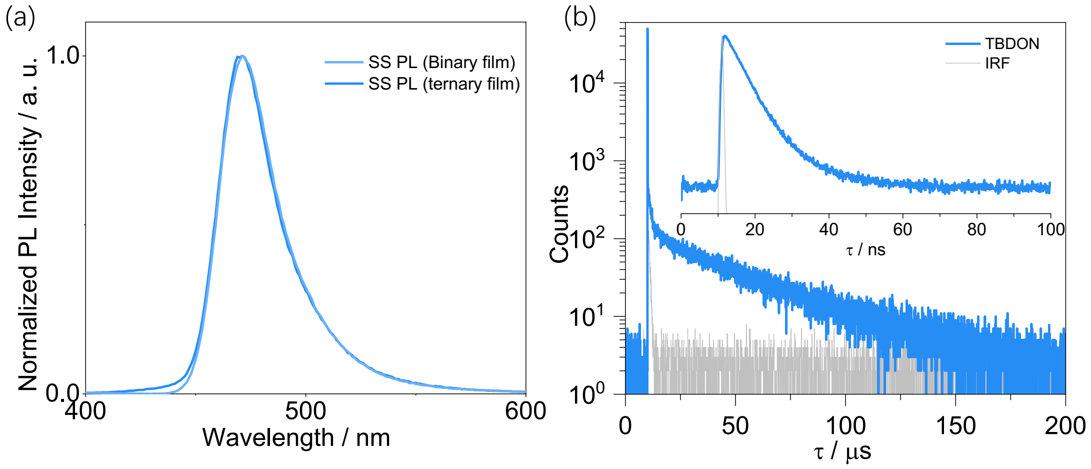

Figure **S27**. (a) Comparation between steady-state PL between the binary film and the ternary films, λ_{exc} = 340 nm. (b) Time-resolved PL decay of the ternary film, λ_{exc} = 379 nm. Binary film: 4 wt% doped film of **TBDON** in **2,6-DCzPPy** film, Ternary film 2 wt% **TBDON**/ 20 wt% **DMAC-DPS** doped in 2,6-DCzPPy film.

Table **S5**. Key parameters of reported devices based on blue MR-TADF OLEDs with emitters containing a multi-boron skeleton.

**Emitters**[a]	λ_{EL} / nm	EQE_{max} / %	**CIE**	**FWHM/ nm**	**References**
**TBDON**	471	24.4/20.2/13.7	0.12,0.16	29	This work
**TBDON**[a]	471	28.1/24.4/17.5	0.12,0.17	30	
**α-3BNMes**	-	1.7/-/-	0.15,0.08	-	29
**α-3BNMes**[a]	443	14.6/10.2/-	0.15, 0.10	49	
**NOBNacene**	412	11.2/-/-	0.18, 0.07	41	30
**MesB-DIDOBNA-N**	402	16.2/3.5/-	0.17, 0.05	21	31
***f*-DOABNA**	445	19.5/15.9/7.5	0.15,0.04	24	32
***v*-DABNA-Mes**	480	22.9/20.3/10.9	0.09,0.21	27	33
***v*-DABNA**	483	26.2/26.2/25.3	0.09,0.27	17	34
***v*-DABNA-F**	468	26.6/25.8/23.4	0.12,0.10	15	
**CzB4-oPh**	490	28.7/27.8/25.8	0.09,0.45	21	35
**DOB2-DABNA-A**	452	24.1/23.3/21.6	0.15,0.05	24	36
**DOB2-DABNA-B-NP**	471	29.1/26.7/24.0	0.12,0.13	23	
**B2**	460	18.3/12.6/-	0.13,0.11	37	37
**DABNA-3B**	475	33.8/30.6/25.1	-	25	38
**BCzBN-3B**	493	42.6/40.4/30.5	-	22	
**DPA-B4**	457	39.2/36.4/28.7	0.150,0.04	14	39
**DPA-B3**	450	37.7/33.7/21.0	0.14,0.05	15	
**Cz-B4**	457	32.1/26.9/16.1	0.14,0.08	26	

[a]In a device with **2,6-DCzPPy** as the assistant dopant.

References

1 M. J. Frisch, G. W. Trucks, H. B. Schlegel, G. E. Scuseria, M. A. Robb, J. R. Cheeseman, G. Scalmani, V. Barone, G. A. Petersson, H. Nakatsuji, X. Li, M. Caricato, A. V. Marenich, J. Bloino, B. G. Janesko, R. Gomperts, B. Mennucci, H. P. Hratchian, J. V. Ortiz, A. F. Izmaylov, J. L. Sonnenberg, Williams, F. Ding, F. Lipparini, F. Egidi, J. Goings, B. Peng, A. Petrone, T. Henderson, D. Ranasinghe, V. G. Zakrzewski, J. Gao, N. Rega, G. Zheng, W. Liang, M. Hada, M. Ehara, K. Toyota, R. Fukuda, J. Hasegawa, M. Ishida, T. Nakajima, Y. Honda, O. Kitao, H. Nakai, T. Vreven, K. Throssell, J. A. Montgomery Jr., J. E. Peralta, F. Ogliaro, M. J. Bearpark, J. J. Heyd, E. N. Brothers, K. N. Kudin, V. N. Staroverov, T. A. Keith, R. Kobayashi, J. Normand, K. Raghavachari, A. P. Rendell, J. C. Burant, S. S. Iyengar, J. Tomasi, M. Cossi, J. M. Millam, M. Klene, C. Adamo, R. Cammi, J. W. Ochterski, R. L. Martin, K. Morokuma, O. Farkas, J. B. Foresman, D. J. Fox, Gaussian 16, Revision C. 01., Gaussian, Inc., Wallingford CT., 2016.2 C. Adamo and V. Barone, *J. Chem. Phys.*, 1999, **110**, 6158–6170.

3 T. H. Dunning Jr., *J. Chem. Phys.*, 1989, **90**, 1007–1023.

4 S. Grimme, *Chem. Phys. Lett.*, 1996, **259**, 128–137.

5 S. Hirata and M. Head-Gordon, *Chem. Phys. Lett.*, 1999, **314**, 291–299.

6 R. Dennington, T. A. Keith and J. M. Millam, *Semichem Inc Shawnee Mission KS.*

7 C. Hättig, *J. Chem. Phys.*, 2003, **118**, 7751–7761.

8 A. Hellweg, S. A. Grün and C. Hättig, *Phys. Chem. Chem. Phys.*, 2008, **10**, 4119–4127.

9 O. S. Lee, E. Zysman-Colman, Digichem (version 6) InSilico Computing, St Andrews, Scotland, 2024.

10 O. Lee, M. Gather, E. Zysman-Colman, ChemRxiv. (Preprint) 2024, 10.26434/chemrxiv-2024-v9vrf

11 N. M. O'Boyle, A. L. Tenderholt and K. M. Langner, *J. Comput. Chem.*, 2008, **29**, 839–845.

12 W. Humphrey, A. Dalke and K. Schulten, *VMD: Visual Molecular Dynamics*, 1996.

13 J. E. Stone, An efficient library for parallel ray tracing and animation, 1998.

14 J. D. Hunter, *Comput. Sci. Eng.*, 2007, **9**, 90–95.

15 N. M. O'Boyle, M. Banck, C. A. James, C. Morley, T. Vandermeersch and G. R. Hutchison, *J. Cheminform.*, 2011, **3**, 33.

16 N. M. O'Boyle, C. Morley and G. R. Hutchison, *Chem. Cent*. J. 2008, **2**, 1-7.

17 X. Gao, S. Bai, D. Fazzi, T. Niehaus, M. Barbatti and W. Thiel, *J. Chem. Theory Comput.*, 2017, **13**, 515–524.

18 N. G. Connelly and W. E. Geiger, *Chem. Rev.* 1996, **96**, 877-910.

19 J. Pommerehne, H. Vestweber, W. Guss, R. F. Mahrt, H. Bässler, M. Porsch and J. Daub, *Adv. Mater.*, 1995, **7**, 551–554.

20 W. H. Melhuish, *J. Phys. Chem.*, 1961, **65**, 229–235.

21 N. C. Greenham, I. D. W. Samuel, G. R. Hayes, R. T. Phillips, Y. A. R. R. Kessener, S. C. Moratti, A. B. Holmes and R. H. Friend, *Chem. Phys. Lett.*, 1995, **241**, 89–96.

22 *CrysAlisPro* v1.171.42.83a Rigaku Oxford Diffraction, Rigaku Corporation, *Tokyo,* Japan, 2023.

23 Sheldrick, G. M. SHELXT – Integrated space-group and crystal structure determination. *Acta Crystallogr., Sect. A: Found. Adv.* **2015**, *71*, 3-8.

24 Sheldrick, G. M. Crystal structure refinement with SHELXL. *Acta Crystallogr., Sect. C: Struct. Chem.* **2015**, *71*, 3-8.

25 Dolomanov, O. V.; Bourhis, L. J.; Gildea, R. J.; Howard, J. A. K.; Puschmann, H. OLEX2: a complete structure solution, refinement and analysis program. *J. Appl. Crystallogr*. **2009**, *42*, 339-341.

26 S. A. Bagnich, A. Rudnick, P. Schroegel, P. Strohriegl and A. Köhler, *Philos. T. R. Soc. A*, **2015**. *373*, 20140446.

27 Y. Tsuchiya, S. Diesing, F. Bencheikh, Y. Wada, P. L. dos Santos, H. Kaji, E. Zysman-Colman, I. D. W. Samuel and C. Adachi, *J. Phys. Chem. A*, 2021, **125**, 8074–8089.

28 S. Diesing, L. Zhang, E. Zysman-Colman and I. D. W. Samuel, *Nature*, 2024, **627**, 747–753.

29 K. Stavrou, S. Madayanad Suresh, D. Hall, A. Danos, N. A. Kukhta, A. M. Z. Slawin, S. Warriner, D. Beljonne, Y. Olivier, A. Monkman and E. Zysman-Colman, *Adv. Opt. Mater.*, 202200688.

30 S. Madayanad Suresh, L. Zhang, D. Hall, C. Si, G. Ricci, T. Matulaitis, A. M. Z Slawin, S. Warriner, Y. Olivier, I. D. W Samuel and E. Zysman-Colman, *Angew. Chem. Int. Ed.*, 2023, **62**, 20221552.

31 S. M. Suresh, L. Zhang, T. Matulaitis, D. Hall, C. Si, G. Ricci, A. M. Z. Slawin, S. Warriner, D. Beljonne, Y. Olivier, I. D. W. Samuel and E. Zysman-Colman, *Adv. Mater.*, **2023**, *35*, 2300997.

32 R. W. Weerasinghe, S. Madayanad Suresh, D. Hall, T. Matulaitis, A. M. Z. Slawin, S. Warriner, Y. T. Lee, C. Y. Chan, Y. Tsuchiya, E. Zysman-Colman and C. Adachi, *Adv. Mater.*, **2024**, *36*, 2402289.

33 S. Oda, B. Kawakami, Y. Yamasaki, R. Matsumoto, M. Yoshioka, D. Fukushima, S. Nakatsuka and T. Hatakeyama, *J. Am. Chem. Soc.*, **2022**, *144*, 106–112.

34 S. Oda, B. Kawakami, M. Horiuchi, Y. Yamasaki, R. Kawasumi and T. Hatakeyama, *Adv. Sci.*, 202205070.

35 Y. Sano, T. Shintani, M. Hayakawa, S. Oda, M. Kondo, T. Matsushita and T. Hatakeyama, *J. Am. Chem. Soc.*, **2023**, *145*, 11504–11511.

36 J. Ochi, Y. Yamasaki, K. Tanaka, Y. Kondo, K. Isayama, S. Oda, M. Kondo and T. Hatakeyama, *Nat. Commun.*, **2024**, *15*, 2361.

37 K. Matsui, S. Oda, K. Yoshiura, K. Nakajima, N. Yasuda and T. Hatakeyama, *J. Am. Chem. Soc.*, **2018**, *140*, 1195–1198.

38 X. Huang, J. Liu, Y. Xu, G. Chen, M. Huang, M. Yu, X. Lv, X. Yin, Y. Zou, J. Miao, X. Cao and C. Yang, *Natl. Sci. Rev.*, **2024**, *11*,115.

39 T. Hua, X. Cao, J. Miao, X. Yin, Z. Chen, Z. Huang and C. Yang, *Nat. Photonics*, **2024**, *18*, 1161.
